# Supplementary material for: Quantifying inequities in COVID-19 vaccine distribution over time by social vulnerability, race and ethnicity, and location: A population-level analysis in St. Louis and Kansas City, Missouri
Source: PLoS Med. 2022 Aug 26;19(8):e1004048. doi: 10.1371/journal.pmed.1004048 (PMC9417193; doi:10.1371/journal.pmed.1004048)
Supplement: S1 Fig — (DOCX) [file pmed.1004048.s001.docx]

**S1 Fig: Cumulative Incidence of Primary COVID-19 Vaccination Series and Booster Completion by Race/Ethnicity and SVI.** Estimates represent 7-day moving averages derived from multiply imputed datasets. Denominators represent the total population greater than or equal to 12 years old. Low SVI indicates zip codes with SVIs less than 0.333, medium SVI indicates SVIs between 0.333 and 0.666, and high SVI indicates SVIs greater than or equal to 0.666. SVI=Social Vulnerability Index.
