# Supplementary material for: Quantifying inequities in COVID-19 vaccine distribution over time by social vulnerability, race and ethnicity, and location: A population-level analysis in St. Louis and Kansas City, Missouri
Source: PLoS Med. 2022 Aug 26;19(8):e1004048. doi: 10.1371/journal.pmed.1004048 (PMC9417193; doi:10.1371/journal.pmed.1004048)
Supplement: S4 Fig — (DOCX) [file pmed.1004048.s004.docx]

**S4 Fig: Rates of Diagnosed Cases and Deaths from COVID-19.** Estimates represent 7-day moving averages derived from multiply imputed data.
