# Supplementary material for: Quantifying inequities in COVID-19 vaccine distribution over time by social vulnerability, race and ethnicity, and location: A population-level analysis in St. Louis and Kansas City, Missouri
Source: PLoS Med. 2022 Aug 26;19(8):e1004048. doi: 10.1371/journal.pmed.1004048 (PMC9417193; doi:10.1371/journal.pmed.1004048)

**S6 Fig: Lorenz Curves of Disparities in COVID-19 Vaccinations over Time – Boosters.** This figure depicts modified Lorenz curve examining disparities in receiving COVID-19 boosters as they have changed over time. The units of analysis are zip codes and they are color-coded by their SVI. The dashed line represents equitable distribution where 50% of vaccinations would be conducting in zip codes accounting for 50% of hospitalizations. Lorenz curves measure disparities in the distribution of COVID-19 vaccinations relative to the total population, diagnosed COVID-19 cases, COVID-19 deaths, and total social vulnerability.

**Boosters**


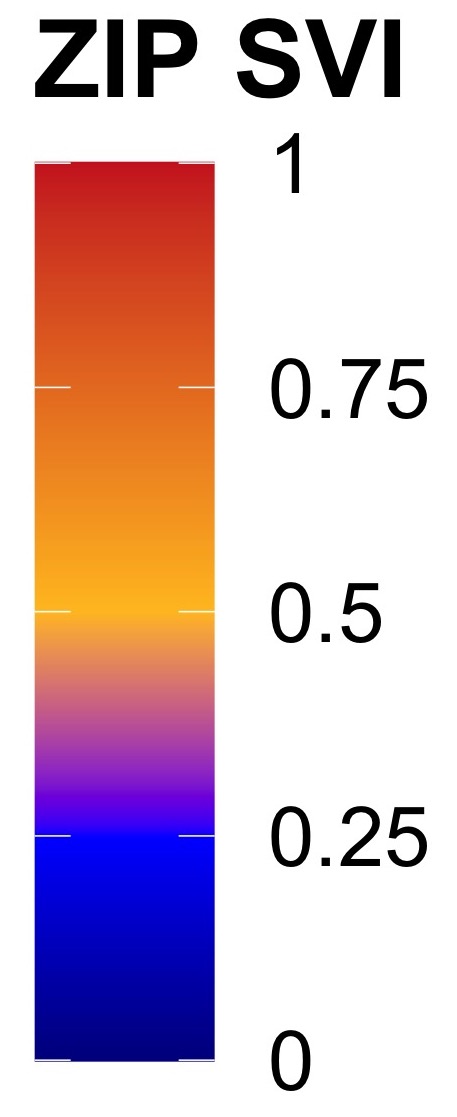

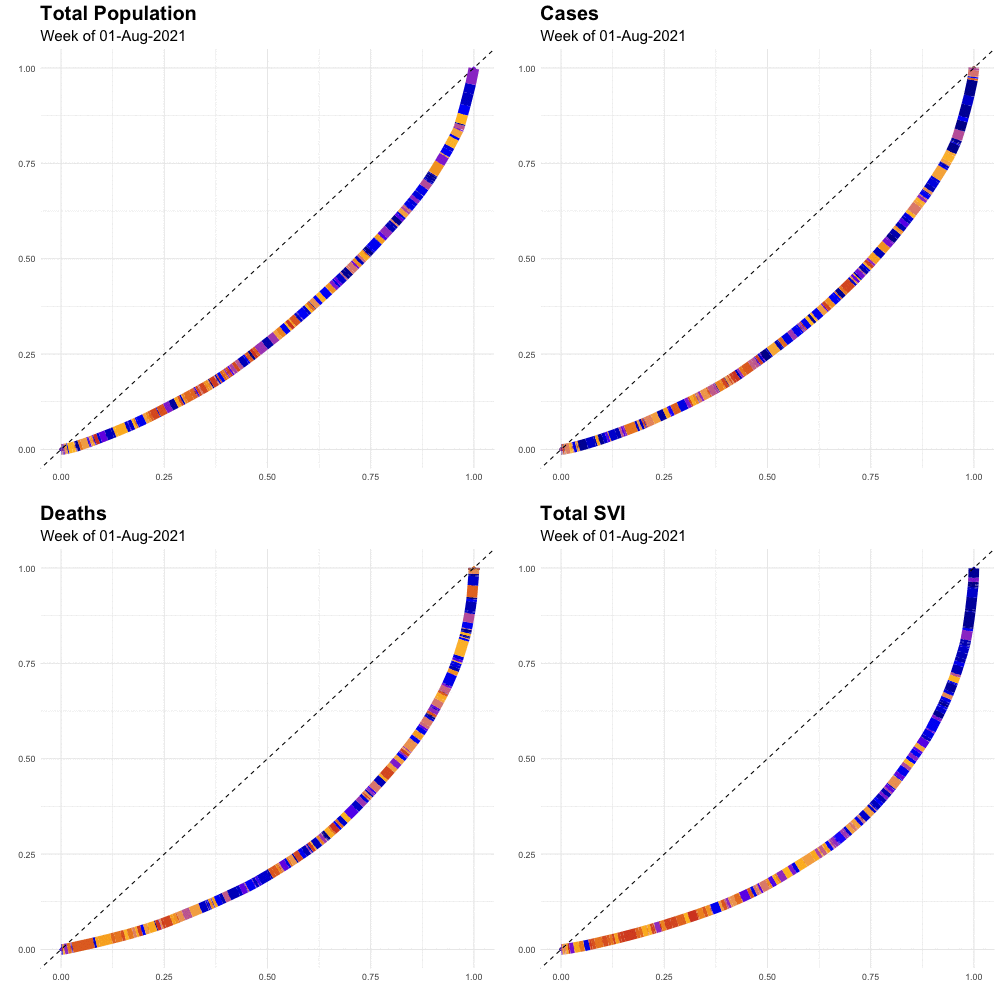

Supplement: S6 Fig — (DOCX) [file pmed.1004048.s006.docx]
