# Supplementary material for: Quantifying inequities in COVID-19 vaccine distribution over time by social vulnerability, race and ethnicity, and location: A population-level analysis in St. Louis and Kansas City, Missouri
Source: PLoS Med. 2022 Aug 26;19(8):e1004048. doi: 10.1371/journal.pmed.1004048 (PMC9417193; doi:10.1371/journal.pmed.1004048)
Supplement: S7 Fig — (DOCX) [file pmed.1004048.s007.docx]

**S7 Fig: Disparities in COVID-19 Primary Vaccine Series and Boosters Among Black, Hispanic, and Asian versus White residents of the same zip code over time.** This figure depicts vaccination rates for the primary series and boosters for Black (Panel A, B), Hispanic (C, D), and Asian (E, F) residents compared to the White residents of the same zip code as they changed over time. Each marker represents a single zip code. Markers are color-coded by the zip code SVI and sized by the total number of vaccines administered in the zip code. The dashed line represents equitable vaccine distribution between racial/communities being compared. Zip codes falling above the dashed line indicates that there was decreased vaccination in Black, Hispanic, or Asian residents as opposed to White residents (and vice versa).


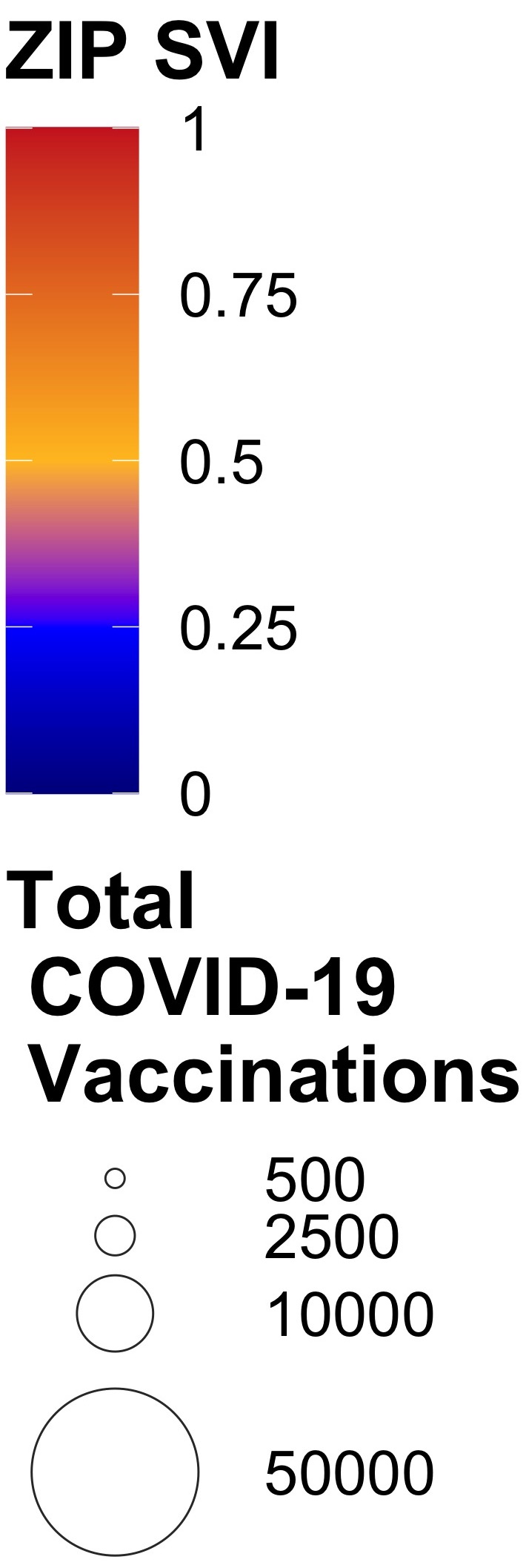

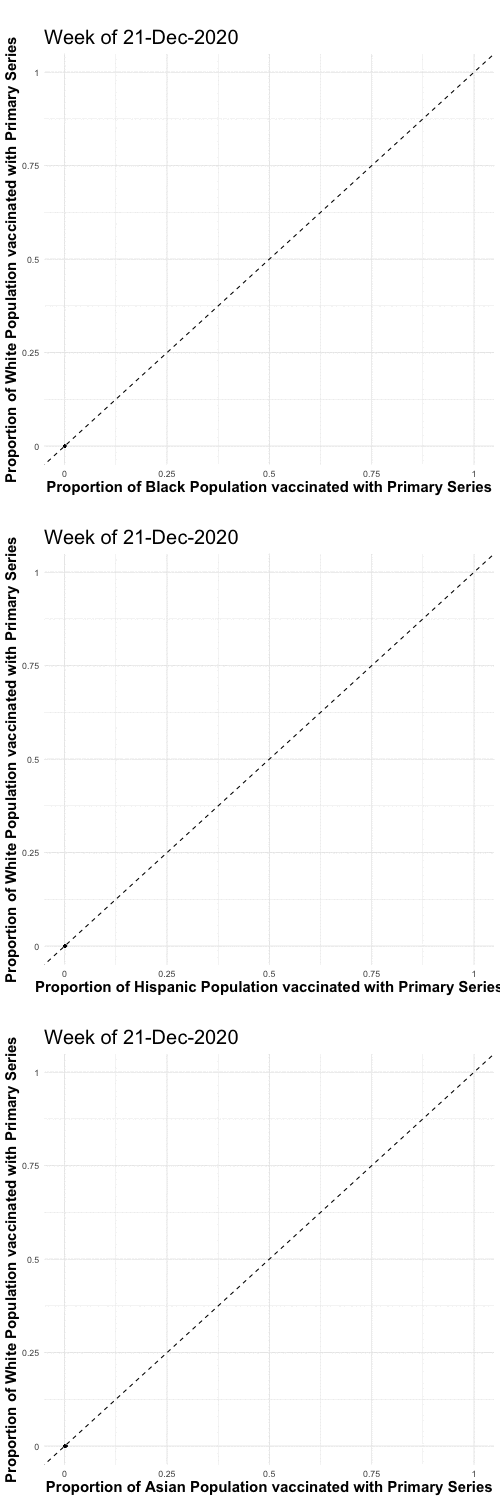


**Primary Series**


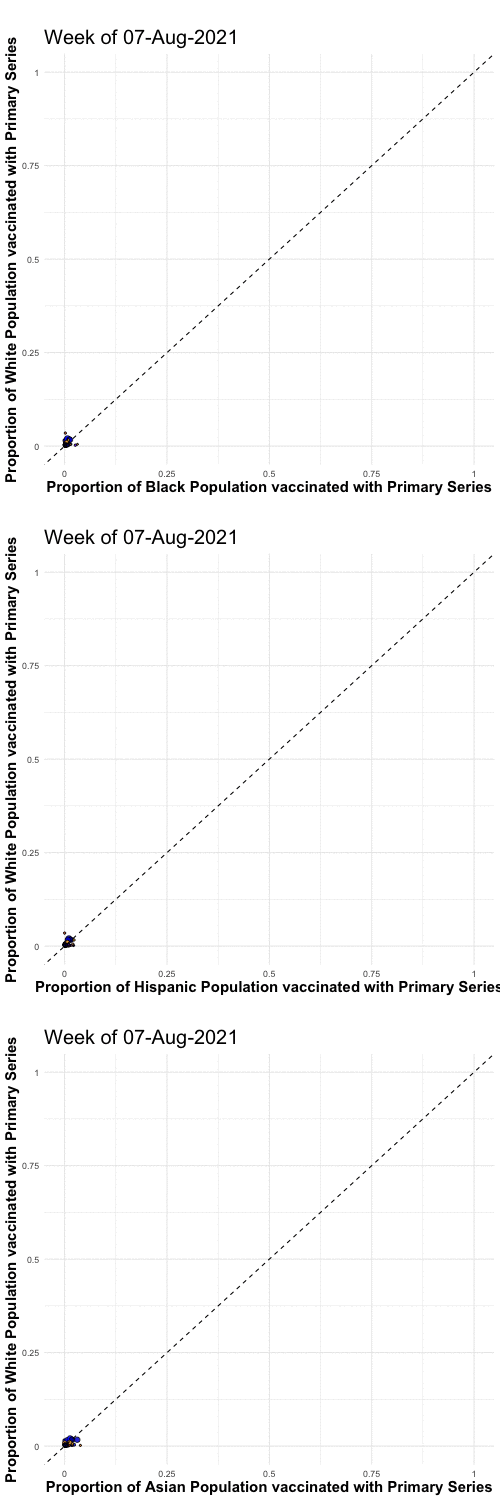


**Booster**
