# Supplementary material for: Quantifying inequities in COVID-19 vaccine distribution over time by social vulnerability, race and ethnicity, and location: A population-level analysis in St. Louis and Kansas City, Missouri
Source: PLoS Med. 2022 Aug 26;19(8):e1004048. doi: 10.1371/journal.pmed.1004048 (PMC9417193; doi:10.1371/journal.pmed.1004048)
Supplement: S1 Table — (DOCX) [file pmed.1004048.s009.docx]

| **S1 Table. Characteristics of individuals completing the primary series** | | | | | | | | | | | |
| --- | --- | --- | --- | --- | --- | --- | --- | --- | --- | --- | --- |
|  | Dec 15, 20 –  Jun 15, 21  (n=1,431,263) | Jun 16, 21 –  Dec 15, 21  (n=311,744) | Dec 16, 21– Feb 15, 22  (n=20,029) |  | Small Volume Health Facility  (n=53,828) | Medium Volume Health Facility  (n=246,975) | Large Volume Health Facility  (n=423,980) | Pharmacy  (n=655,285) | Health Department  (n=304,999) | Employer/  School  (n=39,286) | Other  (n=38,683) |
| Sex*, n (%) |  |  |  |  |  |  |  |  |  |  |  |
| Male | 637,945 (44.6%) | 150,596 (48.3%) | 9,818 (49.0%) |  | 23,807 (44.3%) | 105,955 (42.9%) | 178,302 (42.1%) | 310,090 (47.3%) | 144,176 (47.3%) | 18,301 (47.9%) | 17,278 (44.7%) |
| Female | 792,003 (55.4%) | 161,048 (51.7%) | 10,210 (51.0%) |  | 29,985 (55.7%) | 140,818 (57.1%) | 245,589 (57.9%) | 345,180 (52.7%) | 160,390 (52.7%) | 19,923 (52.1%) | 21,376 (55.3%) |
|  |  |  |  |  |  |  |  |  |  |  |  |
| Age Category*, n (%) |  |  |  |  |  |  |  |  |  |  |  |
| 12-19 years | 84,980  (5.9%) | 69,973 (22.4%) | 5,264 (26.3%) |  | 6,091 (11.3%) | 22,776 (9.2%) | 25,025 (5.9%) | 86,560 (13.2%) | 16,144 (5.3%) | 2,151 (5.5%) | 1,469 (3.8%) |
| 20-34 years | 242,713 (17.0%) | 83,101 (26.7%) | 6,122 (30.6%) |  | 10,102 (18.8%) | 45,624 (18.5%) | 55,251 (13.0%) | 150,337 (22.9%) | 52,151 (17.1%) | 11,495 (29.3%) | 6,976 (18.0%) |
| 35-44 years | 201,922 (14.1%) | 52,272 (16.8%) | 3,211 (16.0%) |  | 7,555 (14.0%) | 36,585 (14.8%) | 47,893 (11.3%) | 106,291 (16.2%) | 47,111 (15.4%) | 6,065 (15.4%) | 5,908 (15.3%) |
| 45-54 years | 203,610 (14.2%) | 44,905 (14.4%) | 2,489 (12.4%) |  | 7,581 (14.1%) | 34,612 (14.0%) | 50,542 (11.9%) | 98,146 (15.0%) | 47,473 (15.6%) | 6,497 (16.5%) | 6,153 (15.9%) |
| 55-64 years | 263,885 (18.4%) | 38,252 (12.3%) | 1,917 (9.6%) |  | 10,300 (19.1%) | 41,263 (16.7%) | 70,712 (16.7%) | 105,912 (16.2%) | 61,508 (20.2%) | 6,975 (17.8%) | 7,384 (19.1%) |
| 65-74 years | 246,781 (17.2%) | 15,863 (5.1%) | 709  (3.5%) |  | 8,156 (15.2%) | 39,860 (16.1%) | 87,748 (20.7%) | 62,689 (9.6%) | 55,506 (18.2%) | 4,022 (10.2%) | 5,372 (13.9%) |
| 75+ years | 187,372 (13.1%) | 7,375  (2.4%) | 318  (1.6%) |  | 4,043 (7.5%) | 26,255 (10.6%) | 86,809 (20.5%) | 45,350 (6.9%) | 25,106 (8.2%) | 2,081 (5.3%) | 5,421 (14.0%) |
|  |  |  |  |  |  |  |  |  |  |  |  |
| Race*, n (%) |  |  |  |  |  |  |  |  |  |  |  |
| Black | 153,724 (11.2%) | 67,342 (22.3%) | 5,454 (28.7%) |  | 9,477 (19.0%) | 28,443 (12.1%) | 51,549 (12.4%) | 80,441 (12.7%) | 40,798 (13.9%) | 7,960 (22.5%) | 7,852 (23.6%) |
| White | 916,653 (66.5%) | 162,831 (53.9%) | 9,654 (50.9%) |  | 31,848 (63.7%) | 156,876 (66.6%) | 276,770 (66.7%) | 390,072 (61.4%) | 192,537 (65.5%) | 21,035 (59.4%) | 20,000 (60.1%) |
| Hispanic | 43,003  (3.1%) | 15,699 (5.2%) | 1,377 (7.3%) |  | 1,632 (3.3%) | 86,32 (3.7%) | 7,798 (1.9%) | 28,621 (4.5%) | 11,171 (3.8%) | 981  (2.8%) | 1,244 (3.7%) |
| Asian | 41,484  (3.0%) | 5,815 (1.9%) | 543  (2.9%) |  | 983  (2.0%) | 5,746 (2.4%) | 10,019 (2.4%) | 18,317 (2.9%) | 10,606 (3.6%) | 1,614 (4.6%) | 557  (1.7%) |
| Other | 222,694 (16.2%) | 50,415 (16.7%) | 1,945 (10.3%) |  | 6,041 (12.1%) | 35,895 (15.2%) | 68,861 (16.6%) | 118,136 (18.6%) | 38,649 (13.2%) | 3,821 (10.8%) | 3,651 (11.0%) |
|  |  |  |  |  |  |  |  |  |  |  |  |
| Median Zip Code SVI, (IQR) | 0.28  (0.16, 0.47) | 0.36  (0.20, 0.57) | 0.41  (0.22, 0.64) |  | 0.31  (0.17, 0.49) | 0.28  (0.16, 0.47) | 0.25  (0.16, 0.47) | 0.31  (0.16, 0.48) | 0.30  (0.16, 0.47) | 0.31  (0.16, 0.51) | 0.31  (0.16, 0.51) |
|  |  |  |  |  |  |  |  |  |  |  |  |
| Vaccine Location Type, n (%) |  |  |  |  | - | - | - | - | - | - | - |
| Small  Volume  Health  Facility | 39,884  (2.8%) | 12,710 (4.1%) | 1,234  (6.2%) |  | - | - | - | - | - | - | - |
| Medium  Volume  Health  Facility | 206,983 (14.5%) | 38,039 (12.2%) | 1,953 (9.8%) |  | - | - | - | - | - | - | - |
| Large  Volume  Health  Facility | 403,437 (28.2%) | 19,722 (6.3%) | 821  (4.1%) |  | - | - | - | - | - | - | - |
| Pharmacy | 420,943 (29.4%) | 219,408 (70.4%) | 14,934 (74.6%) |  | - | - | - | - | - | - | - |
| Health  Department | 287,005 (20.1%) | 17,061 (5.5%) | 933  (4.6%) |  | - | - | - | - | - | - | - |
| Employer/  School | 38,226  (2.7%) | 1,042 (0.3%) | 18  (0.1%) |  | - | - | - | - | - | - | - |
| Other | 34,785  (2.4%) | 3,762 (1.2%) | 136  (0.7%) |  | - | - | - | - | - | - | - |
|  |  |  |  |  |  |  |  |  |  |  |  |
| Primary Series  Vaccine Type, n (%) |  |  |  |  |  |  |  |  |  |  |  |
| J&J | 94,031  (6.6%) | 19,250 (6.2%) | 2,128 (10.6%) |  | 4,797 (8.9%) | 4,822 (2.0%) | 2,089 (0.5%) | 50,363 (7.7%) | 35,603 (11.7%) | 14,762 (37.6%) | 2,973 (7.7%) |
| Moderna | 426,495 (29.8%) | 54,894 (17.6%) | 3,907 (19.5%) |  | 22,625 (42.0%) | 72,317 (29.3%) | 37,240 (8.8%) | 241,090 (36.8%) | 80,430 (26.4%) | 13,062 (33.2%) | 18,532 (47.9%) |
| Pfizer | 910,737 (63.6%) | 237,600 (76.2%) | 13,994 (69.9%) |  | 26,406 (49.1%) | 169,836 (68.8%) | 384,651 (90.7%) | 363,832 (55.5%) | 188,966 (62.0%) | 11,462 (29.2%) | 17,178 (44.4%) |
|  |  |  |  |  |  |  |  |  |  |  |  |
| Booster received, n (%) | 854,371 (59.7%) | 17,913 (5.7%) | 40  (0.2%) |  | 24,030 (44.6%) | 129,502 (52.4%) | 277,948 (65.6%) | 229,025 (35.0%) | 171,447 (56.2%) | 20,408 (51.9%) | 19,964 (51.6%) |
|  |  |  |  |  |  |  |  |  |  |  |  |
| Time Period, n (%) |  |  |  |  |  |  |  |  |  |  |  |
| Dec 15, 20 -  Jun 15, 21 | - | - | - |  | 39,884 (74.1%) | 206,983 (83.8%) | 403,437 (95.2%) | 420,943 (64.2%) | 287,005 (94.1%) | 38,226 (97.3%) | 34,785 (89.9%) |
| Jun 16, 21 -  Dec 15, 21 | - | - | - |  | 12,710 (23.6%) | 38,039 (15.4%) | 19,722 (4.7%) | 219,408 (33.5%) | 17,061 (5.6%) | 1,042 (2.7%) | 3,762 (9.7%) |
| Dec 16, 21 -  Feb 15, 22 | - | - | - |  | 1,234 (2.3%) | 1,953 (0.8%) | 821  (0.2%) | 14,934 (2.3%) | 933 (0.3%) | 18  (<0.1%) | 136  (0.4%) |

Footnote: *Overall Missing values: Sex: 1,866; Race: 64,403; Zip code: 319. Abbreviations: SVI=Social Vulnerability Index; J&J=Johnson and Johnson
