# Supplementary material for: Quantifying inequities in COVID-19 vaccine distribution over time by social vulnerability, race and ethnicity, and location: A population-level analysis in St. Louis and Kansas City, Missouri
Source: PLoS Med. 2022 Aug 26;19(8):e1004048. doi: 10.1371/journal.pmed.1004048 (PMC9417193; doi:10.1371/journal.pmed.1004048)
Supplement: S2 Table — (DOCX) [file pmed.1004048.s010.docx]

| **S2 Table. Characteristics of individuals receiving a booster vaccination** | | | | | | | | | | | | |
| --- | --- | --- | --- | --- | --- | --- | --- | --- | --- | --- | --- | --- |
|  | Dec 16, 21– Jun 15, 21  (n=9,722) | | Jun 16, 21 –  Dec 15, 21  (n=592,768) | Dec 16, 21– Feb 15, 22  (n=269,834) |  | Small Volume Health Facility  (n=41,300) | Medium Volume Health Facility  (n=86,703) | Large Volume Health Facility  (n=71,385) | Pharmacy  (n=610,285) | Health Department  (n=37,460) | Employer/  School  (n=6,469) | Other  (n=18,722) |
| Sex*, n (%) |  |  | |  |  |  |  |  |  |  |  |  |
| Male | 3,973 (40.9%) | 249,258 (42.1%) | | 122,549 (45.4%) |  | 17,711 (42.9%) | 35,920 (41.4%) | 28,705 (40.2%) | 267,374 (43.8%) | 16,168 (43.2%) | 2,772 (43.3%) | 7,130 (38.1%) |
| Female | 5,746 (59.1%) | 343,432 (57.9%) | | 147,227 (54.6%) |  | 23,580 (57.1%) | 50,771 (58.6%) | 42,674 (59.8%) | 342,902 (56.2%) | 21,265 (56.8%) | 3,627 (56.7%) | 11,586 (61.9%) |
|  |  |  | |  |  |  |  |  |  |  |  |  |
| Age Category*, n (%) |  |  | |  |  |  |  |  |  |  |  |  |
| 12-19 years | 257  (2.6%) | 4,714 (0.8%) | | 36,168 (13.4%) |  | 1,916 (4.6%) | 5,169 (6.0%) | 2,236 (3.1%) | 29,902 (4.9%) | 1,359 (3.6%) | 285  (4.4%) | 272  (1.5%) |
| 20-34 years | 1,383 (14.2%) | 58,406 (9.9%) | | 50,627 (18.8%) |  | 4237 (10,.3%) | 10,068 (11.6%) | 8,458 (11.8%) | 79,664 (13.1%) | 3,870 (10.3%) | 2,436 (37.7%) | 1,683 (9.0%) |
| 35-44 years | 1,299 (13.4%) | 69,830 (11.8%) | | 40,566 (15.0%) |  | 4,305 (10.4%) | 11,764 (13.6%) | 8,699 (12.2%) | 79,170 (13.0%) | 4,864 (13.0%) | 1,097 (17.0%) | 1,796 (9.6%) |
| 45-54 years | 1,458 (15.0%) | 73,821 (12.5%) | | 43,300 (16.0%) |  | 5,233 (12.7%) | 12,266 (14.1%) | 9,442 (13.2%) | 83,073 (13.6%) | 5,115 (13.7%) | 1,050 (16.2%) | 2,400 (12.8%) |
| 55-64 years | 1,913 (19.7%) | 116,013 (19.6%) | | 51,941 (19.2%) |  | 8,371 (20.3%) | 16,980 (19.6%) | 13,322 (18.7%) | 118,813 (19.5%) | 7,923 (21.2%) | 1,003 (15.5%) | 3455 (18,.5%) |
| 65-74 years | 1,866 (19.2%) | 150,294 (25.4%) | | 29,826 (11.1%) |  | 8,763 (21.2%) | 17,338 (20.0%) | 16,127 (22.6%) | 128,062 (21.0%) | 8,299 (22.2%) | 426  (6.6%) | 2,971 (15.9%) |
| 75+ years | 1,546 (15.9%) | 119,690 (20.2%) | | 17,406 (6.5%) |  | 8,475 (20.5%) | 13,118 (15.1%) | 13,101 (18.4%) | 91,601 (15.0%) | 6,030 (16.1%) | 172  (2.7%) | 6,145 (32.8%) |
|  |  |  | |  |  |  |  |  |  |  |  |  |
| Race*, n (%) |  |  | |  |  |  |  |  |  |  |  |  |
| Black | 1,079 (11.3%) | 50,098 (8.6%) | | 33,387 (12.6%) |  | 7,073 (17.4%) | 12,227 (14.3%) | ,9290 (13.2%) | 46,383 (7.7%) | 6,514 (18.4%) | 591  (10.5%) | 2,486 (14.5%) |
| White | 6,102 (63.9%) | 390,295 (67.1%) | | 173,014 (65.3%) |  | 25,989 (64.0%) | 51,780 (60.6%) | 47,164 (66.9%) | 409,591 (68.1%) | 21,900 (61.8%) | 3,767 (66.8%) | 9,220 (53.9%) |
| Hispanic | 260  (2.7%) | 11,318 (1.9%) | | 8,516 (3.2%) |  | 808  (2.0%) | 1,815 (2.1%) | 956  (1.4%) | 14,519 (2.4%) | 1,369 (3.9%) | 177  (3.1%) | 450  (2.6%) |
| Asian | 206  (2.2%) | 13,512 (2.3%) | | 9,693 (3.7%) |  | 954  (2.4%) | 2,289 (2.7%) | 1,856 (2.6%) | 1,7050 (2.8%) | 607  (1.7%) | 473  (8.4%) | 182  (1.1%) |
| Other | 1,895 (19.9%) | 116,689 (20.1%) | | 40,534 (15.3%) |  | 5,764 (14.2%) | 17,335 (20.3%) | 11,262 (16.0%) | 114,303 (19.0%) | 5,066 (14.3%) | 630  (11.2%) | 4,758 (27.8%) |
|  |  |  | |  |  |  |  |  |  |  |  |  |
| Median Zip Code SVI, (IQR) | 0.25  (0.16, 0.47) | 0.25  (0.16, 0.46) | | 0.28  (0.16, 0.47) |  | 0.30  (0.16, 0.48) | 0.23  (0.15, 0.47) | 0.27  (0.16, 0.46) | 0.25  (0.16, 0.46) | 0.37  (0.20, 0.49) | 0.25  (0.15, 0.45) | 0.27  (0.16, 0.48) |
|  |  |  | |  |  |  |  |  |  |  |  |  |
| Vaccine Location Type, n (%) |  |  | |  |  |  |  |  |  |  |  |  |
| Small  Volume  Health  Facility | 587  (6.0%) | 27,144 (4.6%) | | 13,569 (5.0%) |  | - | - | - | - | - | - | - |
| Medium  Volume  Health  Facility | 1,031 (10.6%) | 57,545 (9.7%) | | 28,127 (10.4%) |  | - | - | - | - | - | - | - |
| Large  Volume  Health  Facility | 950  (9.8%) | 53,778 (9.1%) | | 16,657 (6.2%) |  | - | - | - | - | - | - | - |
| Pharmacy | 2,955 (30.4%) | 410,173 (69.2%) | | 197,157 (73.1%) |  | - | - | - | - | - | - | - |
| Health  Department | 2,029 (20.9%) | 25,630 (4.3%) | | 9,801  (3.6%) |  | - | - | - | - | - | - | - |
| Employer/  School | 277  (2.8%) | 4,575  (0.8%) | | 1,617  (0.6%) |  | - | - | - | - | - | - | - |
| Other | 1,893 (19.5%) | 13,923 (2.3%) | | 2,906  (1.1%) |  | - | - | - | - | - | - | - |
|  |  |  | |  |  |  |  |  |  |  |  |  |
| Booster Vaccine Type, n (%) |  |  | |  |  |  |  |  |  |  |  |  |
| J&J | 675  (6.9%) | 6,054  (1.0%) | | 2,072  (0.8%) |  | 162  (0.4%) | 262  (0.3%) | 199  (0.3%) | 6,274  (1.0%) | 1,334  (3.6%) | 181  (2.8%) | 389  (2.1%) |
| Moderna | 3,622 (37.3%) | 204,521 (34.5%) | | 85,666 (31.7%) |  | 5,830 (14.1%) | 10,808 (12.5%) | 2,634 (3.7%) | 246,608 (40.4%) | 13,281 (35.5%) | 1,946 (30.1%) | 12,702 (67.8%) |
| Pfizer | 5,425 (55.8%) | 382,193 (64.5%) | | 182,096 (67.5%) |  | 35,308 (85.5%) | 75,633 (87.2%) | 68,552 (96.0%) | 357,403 (58.6%) | 22,845 (61.0%) | 4,342 (67.1%) | 5,631 (30.1%) |
|  |  |  | |  |  |  |  |  |  |  |  |  |
| Time Period, n (%) |  |  | |  |  |  |  |  |  |  |  |  |
| Dec 15, 20 –  Jun 15, 21 | - | - | | - |  | 587  (1.4%) | 1,031  (1.2%) | 950  (1.3%) | 2,955  (0.5%) | 2,029  (5.4%) | 277  (4.3%) | 1,893 (10.1%) |
| Jun 16, 21  Dec 15, 21 | - | - | | - |  | 27,144 (65.7%) | 57,545 (66.4%) | 53,778 (75.3%) | 410,173 (67.2%) | 25,630 (68.4%) | 4,575 (70.7%) | 13,923 (74.4%) |
| Dec 16, 21 –  Feb 15, 22 | - | - | | - |  | 13,569 (32.9%) | 28,127 (32.4%) | 16,657 (23.3%) | 197,157 (32.3%) | 9,801 (26.2%) | 1,617 (25.0%) | 2,906 (15.5%) |

Footnote: *Overall Missing values: Sex: 139; Race/Ethnicity: 15,726; Zip code: 124. Abbreviations: SVI=Social Vulnerability Index; J&J=Johnson and Johnson
