# Supplementary material for: Quantifying inequities in COVID-19 vaccine distribution over time by social vulnerability, race and ethnicity, and location: A population-level analysis in St. Louis and Kansas City, Missouri
Source: PLoS Med. 2022 Aug 26;19(8):e1004048. doi: 10.1371/journal.pmed.1004048 (PMC9417193; doi:10.1371/journal.pmed.1004048)
Supplement: S3 Table — (DOCX) [file pmed.1004048.s011.docx]

| **S3 Table. Rates of Initiating/Completing Primary Vaccine Series and Boosters by race/ethnicity and SVI** | | | | |
| --- | --- | --- | --- | --- |
|  |  | Primary Series Started (%) | Primary Series Complete (%) | Booster  Complete (%) |
| Black | Low SVI | 69.2 | 58.6 | 24.0 |
|  | Medium SVI | 58.0 | 48.8 | 18.5 |
|  | High SVI | 54.4 | 45.0 | 15.1 |
| White | Low SVI | 67.3 | 59.5 | 31.6 |
|  | Medium SVI | 59.5 | 52.4 | 25.3 |
|  | High SVI | 52.6 | 45.1 | 19.5 |
| Hispanic | Low SVI | 70.0 | 56.7 | 22.5 |
|  | Medium SVI | 67.3 | 52.9 | 16.2 |
|  | High SVI | 67.8 | 50.7 | 10.5 |
| Asian | Low SVI | 77.9 | 64.2 | 32.4 |
|  | Medium SVI | 76.2 | 61.3 | 27.9 |
|  | High SVI | 100 | 80.5 | 29.7 |

Footnote: As of February 15, 2022
