# Supplementary material for: Quantifying inequities in COVID-19 vaccine distribution over time by social vulnerability, race and ethnicity, and location: A population-level analysis in St. Louis and Kansas City, Missouri
Source: PLoS Med. 2022 Aug 26;19(8):e1004048. doi: 10.1371/journal.pmed.1004048 (PMC9417193; doi:10.1371/journal.pmed.1004048)
Supplement: S4 Table — (DOCX) [file pmed.1004048.s012.docx]

| **S4 Table. Characteristics of Zip Codes by Quartile of Lorenz Curve – Number of COVID-19 Vaccinations relative to Total Population** | | | | | | | | | | | | |
| --- | --- | --- | --- | --- | --- | --- | --- | --- | --- | --- | --- | --- |
|  | **Primary Series** | | | | |  | | **Booster** | | | | |
|  | Lowest Quartile (n=81) | Second Quartile  (n=46) | Third Quartile  (n=35) | Highest Quartile  (n=50) | p-value | |  | Lowest Quartile (n=77) | Second Quartile  (n=53) | Third  Quartile  (n=37) | Highest Quartile  (n=45) | p-value |
|  |  |  |  |  |  | |  |  |  |  |  |  |
| Percent of COVID-19 Vaccinations | 19.3 | 23.4 | 26.6 | 30.7 | <0.001 | |  | 15.6 | 21.9 | 27.1 | 35.5 | <0.001 |
| Percent of Total Population | 24.8 | 24.8 | 25.2 | 25.3 | <0.001 | |  | 24.9 | 24.9 | 24.8 | 25.4 | <0.001 |
|  |  |  |  |  |  | |  |  |  |  |  |  |
| Total Population, median (IQR) | 7,785  (2,117, 15,989) | 15,316  (5,846, 26,468) | 20,455  (7,418, 31,010) | 12,316  (3,038, 22,133) | <0.001 | |  | 8,580  (2,754, 15,989) | 13,419  (3,282, 24,516) | 18,024  (5,981, 31,010) | 17,707  (4,881, 26,768) | 0.01 |
| Percent Black, median (IQR) | 3.8  (0.6, 45.1) | 4.8  (0.3, 34.0) | 5.0  (1.9, 7.9) | 3.8  (0.9, 10.3) | 0.5 | |  | 9.6  (0.9, 51.5) | 2.5  (0.1, 10.1) | 5.9  (2.7, 8.2) | 3.3  (1.1, 8.4) | 0.005 |
| Zip Codes greater than 25% Black, n (%) | 27  (33%) | 14  (30%) | 3  (9%) | 4  (8%) | <0.001 | |  | 32  (42%) | 10  (19%) | 2  (5%) | 4  (9%) | <0.001 |
|  |  |  |  |  |  | |  |  |  |  |  |  |
| Percent Male, median (IQR) | 48.9  (47.1, 50.9) | 49.1  (48.0, 50.3) | 48.7  (48.1, 50.2) | 48.5  (47.2, 50.5) | 0.93 | |  | 48.6  (46.9, 50.6) | 49.3  (48.2, 50.9) | 48.8  (47.4, 50.2) | 48.6  (47.4, 50.5) | 0.31 |
| Median Age, median (IQR) | 37.8  (33.8, 41.9) | 38.5  (35.6, 40.8) | 38.3  (35.7, 40.4) | 40.8  (35.6, 43.6) | 0.14 | |  | 36.9  (33.6, 40.6) | 38.6  (35.7, 41.3) | 39.1  (36.5, 41.8) | 40.7  (35.0, 43.6) | 0.021 |
| Average Household Size, median (IQR) | 3.1  (3.0, 3.3) | 3.1  (3.0, 3.2) | 3.0  (2.9, 3.2) | 3.0  (2.9, 3.1) | 0.058 | |  | 3.2  (3.0, 3.3) | 3.0  (2.9, 3.1) | 3.1  (3.0, 3.2) | 3.0  (2.9, 3.1) | <0.001 |
|  |  |  |  |  |  | |  |  |  |  |  |  |
| Median Income ($), median (IQR) | 46,494  (36,074, 60,131) | 60,814  (46,915, 75,288) | 68,841  (57,215, 79,930) | 76,236  (57,946, 99,341) | <0.001 | |  | 43,511  (30,724, 55,363) | 61,420  (50,681, 74,864) | 73,397  (61,061, 88,919) | 76,935  (59,029, 99,525) | <0.001 |
| Percent below poverty line, median (IQR) | 12.1  (4.8, 17.5) | 6.6  (4.1, 12.3) | 5.4  (2.9, 7.6) | 3.0  (1.6, 6.6) | <0.001 | |  | 13.6  (8.6, 23.3) | 6.0  (3.7, 9.7) | 4.3  (2.6, 7.0) | 2.9  (1.5, 5.3) | <0.001 |
| Percent with no health insurance, median (IQR) | 10.9  (7.6, 15.6) | 7.8  (5.9, 12.6) | 6.1  (4.4, 8.6) | 4.3  (2.6, 8.1) | <0.001 | |  | 12.7  (8.8, 16.7) | 7.5  (5.7, 10.0) | 5.5  (4.3, 7.2) | 3.7  (2.4, 6.5) | <0.001 |
|  |  |  |  |  |  | |  |  |  |  |  |  |
| Percent in Healthcare Industry, median (IQR) | 21.1  (17.4, 24.7) | 21.8  (19.6, 24.6) | 22.5  (20.7, 24.6) | 24.2  (19.5, 27.4) | 0.18 | |  | 21.1  (17.4, 24.7) | 21.5  (19.4, 23.8) | 23.3  (21.9, 24.7) | 25.0  (19.5, 27.7) | 0.038 |
| Percent in Service Industry, median (IQR) | 19.3  (14.0, 23.6) | 17.7  (14.7, 21.1) | 15.1  (12.4, 17.0) | 11.4  (8.7, 15.4) | <0.001 | |  | 20.1  (15.1, 27.2) | 16.5  (13.9, 19.4) | 14.4  (11.1, 16.0) | 11.3  (8.7, 15.1) | <0.001 |
| Percent Commuting via Public Transportation, median (IQR) | 0.3  (0.0, 4.1) | 0.4  (0.0, 2.5) | 0.3  (0.1, 1.2) | 0.5  (0.0, 2.0) | 0.98 | |  | 0.6  (0.0, 7.4) | 0.3  (0.0, 1.5) | 0.2  (0.0, 0.7) | 0.5  (0.1, 2.0) | 0.094 |
| Percent Working from Home, median (IQR) | 3.7  (2.2, 5.3) | 3.5  (2.7, 4.9) | 4.7  (3.6, 7.0) | 6.0  (4.2, 7.7) | <0.001 | |  | 3.4  (2.1, 4.9) | 4.1  (2.9, 5.6) | 4.7  (3.6, 6.6) | 6.5  (4.2, 8.1) | <0.001 |
|  |  |  |  |  |  | |  |  |  |  |  |  |
| Cases per 100,000 population, median (IQR) | 19,506  (16,014, 21,781) | 21,422  (19,674, 23,687) | 21,630  (19,644, 23,244) | 22,295  (19,954, 25,078) | <0.001 | |  | 19,808  (16,014, 22,050) | 21,851  (19,585, 23,521) | 21,765  (19,884, 23,761) | 21,087  (19,189, 24,250) | <0.001 |
| Deaths per 100,000 population, median (IQR) | 217  (122, 343) | 219  (143, 288) | 188  (122, 259) | 198  (99, 330) | 0.72 | |  | 241  (130, 345) | 186  (133, 288) | 199  (122, 240) | 184  (91, 270) | 0.29 |
| Vaccine Locations per 10,000 population, median (IQR) | 2.6  (1.6, 3.0) | 3.3  (2.0, 4.5) | 3.7  (2.2, 4.8) | 3.9  (2.4, 6.2) | 0.004 | |  | 2.4  (1.7, 3.1) | 3.3  (2.0, 4.0) | 4.0  (2.8, 5.5) | 3.7  (2.4, 5.7) | 0.003 |
|  |  |  |  |  |  | |  |  |  |  |  |  |
|  |  |  |  |  |  | |  |  |  |  |  |  |
| Overall SVI, median (IQR) | 48.8  (34.3, 70.8) | 42.4  (24.7, 50.9) | 28.5  (15.9, 42.8) | 19.6  (12.2, 30.4) | <0.001 | |  | 55.9  (42.1, 79.6) | 32.7  (21.5, 46.2) | 28.5  (16.1, 45.4) | 16.4  (11.9, 25.0) | <0.001 |
| Socioeconomic  theme, median  (IQR) | 54.6  (39.4, 74.3) | 45.0  (25.3, 66.4) | 32.2  (17.6, 41.6) | 19.1  (10.6, 35.1) | <0.001 | |  | 66.5  (45.5, 79.8) | 39.3  (25.3, 50.1) | 25.5  (15.6, 40.4) | 17.9  (10.2, 34.5) | <0.001 |
| Household  Composition  theme, median  (IQR) | 68.3  (45.8, 82.7) | 58.1  (44.8, 71.4) | 45.0  (31.4, 56.4) | 34.1  (23.7, 46.3) | <0.001 | |  | 72.4  (55.1, 83.4) | 51.6  (35.7, 62.9) | 45.0  (31.4, 59.4) | 33.9  (20.9, 42.5) | <0.001 |
| Minority  Status/Language  Theme, median  (IQR) | 20.7  (8.7, 55.0) | 27.1  (14.5, 53.9) | 31.2  (19.4, 40.4) | 26.8  (21.4, 41.1) | 0.87 | |  | 37.9  (10.8, 58.1) | 19.4  (9.7, 40.5) | 32.5  (23.5, 44.1) | 26.7  (21.4, 37.0) | 0.014 |
| Infrastructure  theme, median  (IQR) | 52.6  (34.7, 65.9) | 36.6  (26.6, 52.1) | 35.7  (22.2, 50.6) | 29.4  (16.5, 42.1) | <0.001 | |  | 59.1  (38.7, 69.0) | 36.6  (26.1, 48.6) | 29.3  (21.8, 43.3) | 28.5  (16.4, 38.6) | <0.001 |
|  |  |  |  |  |  | |  |  |  |  |  |  |
| Percent receiving at least one vaccine dose, median (IQR) | 42.0  (37.8, 44.8) | 51.4  (49.7, 52.8) | 57.7  (56.6, 58.9) | 67.6  (63.0, 71.4) | <0.001 | |  | 15.9  (14.1, 18.6) | 23.7  (22.3, 24.9) | 29.7  (28.5, 30.7) | 38.4  (34.9, 41.9) | <0.001 |
|  |  |  |  |  |  | |  |  |  |  |  |  |
| Percent Vaccinated at: |  |  |  |  |  | |  |  |  |  |  |  |
| Small Volume  Health Facility,  median (IQR) | 3.7  (3.0, 4.9) | 3.0  (2.7, 3.6) | 2.7  (2.5, 3.4) | 2.5  (2.3, 3.0) | <0.001 | |  | 3.4  (2.5, 4.2) | 3.1  (2.4, 3.7) | 2.6  (2.2, 3.1) | 2.4  (2.2, 2.7) | <0.001 |
| Medium Volume  Health Facility,  median (IQR) | 12.5  (9.3, 16.4) | 13.9  (9.8, 17.9) | 12.7  (10.6, 19.1) | 12.1  (10.2, 13.9) | 0.42 | |  | 14.0  (9.8, 19.5) | 13.1  (10.5, 20.1) | 13.2  (11.8, 22.9) | 12.0  (10.7, 13.5) | 0.31 |
| Large Volume  Health Facility,  median (IQR) | 13.7  (8.4, 22.6) | 21.1  (12.8, 26.9) | 25.2  (14.8, 30.4) | 30.2  (23.9, 34.8) | <0.001 | |  | 20.8  (11.8, 32.7) | 28.7  (14.1, 36.3) | 31.2  (16.7, 38.6) | 39.1  (34.4, 42.1) | <0.001 |
| Pharmacy,  median (IQR) | 40.9  (36.4, 47.9) | 41.1  (35.1, 47.1) | 36.7  (32.3, 44.1) | 31.5  (28.0, 37.4) | <0.001 | |  | 28.1  (23.4, 34.5) | 28.2  (24.7, 32.9) | 28.2  (22.7, 33.3) | 23.3  (22.2, 26.0) | 0.005 |
| Health  department,  median (IQR) | 18.7  (14.5, 23.5) | 17.0  (15.2, 20.3) | 17.5  (15.2, 19.3) | 16.7  (15.1, 19.8) | 0.26 | |  | 22.5  (17.1, 27.9) | 22.0  (17.6, 24.8) | 21.3  (16.8, 23.5) | 17.0  (14.9, 21.6) | <0.001 |
| Employer/school,  median (IQR) | 0.8  (0.4, 3.0) | 0.9  (0.6, 2.1) | 1.6  (0.9, 2.4) | 2.0  (1.2, 3.2) | <0.001 | |  | 0.9  (0.5, 3.4) | 1.2  (0.6, 2.2) | 1.2  (0.8, 2.0) | 2.2  (1.7, 3.6) | 0.002 |
| Other,  median (IQR) | 2.0  (1.3, 2.9) | 1.7  (1.4, 2.5) | 2.0  (1.7, 2.3) | 1.9  (1.5, 2.4) | 0.83 | |  | 1.9  (1.2, 2.8) | 2.0  (1.5, 2.6) | 2.0  (1.5, 2.4) | 1.9  (1.6, 2.6) | 0.97 |
|  |  |  |  |  |  | |  |  |  |  |  |  |
| Percent of Vaccinated Receiving: |  |  |  |  |  | |  |  |  |  |  |  |
| J&J,  median (IQR) | 7.5  (6.0, 8.6) | 7.2  (5.7, 8.2) | 6.4  (5.2, 7.3) | 5.5  (4.9, 7.4) | 0.004 | |  | 7.3  (4.9, 8.9) | 6.7  (5.0, 7.6) | 4.6  (4.1, 6.8) | 4.7  (3.9, 6.6) | <0.001 |
| Moderna,  median (IQR) | 59.8  (53.2, 64.3) | 64.2  (58.5, 68.2) | 66.9  (64.4, 68.7) | 68.5  (64.1, 71.0) | <0.001 | |  | 55.7  (51.5, 60.3) | 59.8  (54.9, 65.7) | 65.8  (62.7, 67.9) | 68.2  (64.2, 71.2) | <0.001 |
| Pfizer,  median (IQR) | 31.9  (28.1, 39.6) | 28.0  (25.3, 32.9) | 27.1  (24.9, 28.3) | 25.6  (23.4, 27.6) | <0.001 | |  | 36.4  (31.8, 42.1) | 32.0  (27.8, 38.8) | 29.3  (26.7, 33.1) | 25.5  (24.3, 28.1) | <0.001 |
|  |  |  |  |  |  | |  |  |  |  |  |  |
| Region |  |  |  |  |  | |  |  |  |  |  |  |
| Kansas City, n (%) | 36  (44.4%) | 22  (47.8%) | 16  (45.7%) | 14  (28.0%) | 0.17 | |  | 32  (41.6%) | 26  (49.1%) | 20  (54.1%) | 10  (22.2%) | 0.015 |
| St. Louis, n (%) | 45  (55.6%) | 24  (52.2%) | 19  (54.3%) | 36  (72.0%) |  |  |  | 45  (58.4%) | 27  (50.9%) | 17  (45.9%) | 35  (77.8%) |  |

*Notes*: Each quartile corresponds to successive segments of the Lorenz curve so that each quartile contains sufficient consecutive zip codes to account for 25% of the total population. Lorenz curve-based quartiles were generated by first sorting zip codes by their ratio of COVID-19 vaccinations to total population and splitting them such that each quartile accounted for 25% of the overall population. Thus, the first quartile represents zip codes on the leftmost side of the curve (i.e., have the lowest ratio of COVID-19 vaccinations to population) and the last quartile represents the zip codes on the rightmost side of the curve (i.e., have the highest ratio of COVID-19 vaccination to population). P-values were generated based on Kruskal-Wallis tests to assess differences between quartiles. Abbreviations: IQR=interquartile range; SVI=Social Vulnerability Index; J&J=Johnson and Johnson.
