# Supplementary material for: Quantifying inequities in COVID-19 vaccine distribution over time by social vulnerability, race and ethnicity, and location: A population-level analysis in St. Louis and Kansas City, Missouri
Source: PLoS Med. 2022 Aug 26;19(8):e1004048. doi: 10.1371/journal.pmed.1004048 (PMC9417193; doi:10.1371/journal.pmed.1004048)
Supplement: S5 Table — (DOCX) [file pmed.1004048.s013.docx]

| **S5 Table. Characteristics of Zip Codes by Quartile of Lorenz Curve – Number of COVID-19 Vaccinations relative to Diagnosed COVID-19 Cases** | | | | | | | | | | | |  |
| --- | --- | --- | --- | --- | --- | --- | --- | --- | --- | --- | --- | --- |
|  | **Primary Series** | | | | |  | **Booster** | | | | | |
|  | Lowest Quartile (n=79) | Second Quartile  (n=55) | Third Quartile  (n=41) | Highest Quartile  (n=93) | p-value |  | Lowest Quartile (n=79) | Second Quartile  (n=62) | Third Quartile  (n=42) | Highest Quartile  (n=78) | p-value | |
|  |  |  |  |  |  |  |  |  |  |  |  | |
| Percent of COVID-19 Vaccinations | 18.1 | 22.0 | 24.9 | 35.0 | <0.001 |  | 15.0 | 20.9 | 25.9 | 38.2 | <0.001 | |
| Percent of Diagnosed Cases | 24.6 | 24.7 | 24.7 | 26.0 | <0.001 |  | 24.8 | 25.2 | 24.9 | 25.1 | <0.001 | |
|  |  |  |  |  |  |  |  |  |  |  |  | |
| Total Population, median (IQR) | 7,549  (1,916, 16,794) | 11,292  (3,022, 22,208) | 17,517  (9,074, 30,480) | 17,037  (5,894, 26,451) | 0.007 |  | 8,926  (2,101, 16,392) | 9,139  (2,728, 22,738) | 17,524  (5,325, 34,213) | 17,866  (7,878, 26,610) | 0.006 | |
| Percent Black, median (IQR) | 2.9  (0.5, 32.6) | 3.8  (0.3, 12.5) | 5.5  (1.7, 20.6) | 6.0  (1.9, 11.2) | 0.88 |  | 4.6  (0.6, 62.0) | 3.9  (0.2, 25.7) | 4.6  (1.8, 11.1) | 5.3  (1.8, 8.9) | 0.5 | |
| Zip Codes greater than 25% Black, n (%) | 19  (29%) | 13  (25%) | 7  (19%) | 9  (16%) | 0.32 |  | 25  (37%) | 14  (25%) | 4  (11%) | 5  (10%) | 0.001 | |
|  |  |  |  |  |  |  |  |  |  |  |  | |
| Percent Male, median (IQR) | 49.2  (47.1, 50.5) | 48.9  (48.1, 50.6) | 48.8  (47.9, 49.5) | 48.6  (47.4, 50.4) | 0.87 |  | 48.9  (47.0, 50.7) | 49.4  (48.0, 51.0) | 48.4  (47.5, 49.5) | 48.7  (47.3, 50.2) | 0.27 | |
| Median Age, median (IQR) | 36.8  (34.1, 40.6) | 38.3  (36.5, 41.3) | 39.6  (35.8, 41.8) | 40.3  (35.6, 43.7) | 0.042 |  | 36.8  (33.7, 40.0) | 38.5  (35.5, 41.5) | 38.6  (35.8, 41.8) | 40.8  (37.0, 46.6) | 0.004 | |
| Average Household Size, median (IQR) | 3.2  (3.0, 3.3) | 3.1  (3.0, 3.2) | 3.0  (2.9, 3.1) | 3.0  (2.8, 3.1) | 0.003 |  | 3.2  (3.0, 3.3) | 3.0  (2.9, 3.2) | 3.1  (3.0, 3.1) | 3.0  (2.9, 3.1) | 0.002 | |
|  |  |  |  |  |  |  |  |  |  |  |  | |
| Median Income ($), median (IQR) | 50,681  (38,682, 64,390) | 56,250  (45,663, 73,458) | 63,896  (48,105, 85,259) | 69,541  (54,362, 95,261) | <0.001 |  | 45,325  (30,658, 60,730) | 54,499  (44,776, 74,386) | 73,082  (60,036, 92,430) | 75,434  (56,614, 102,526) | <0.001 | |
| Percent below poverty line, median (IQR) | 9.6  (4.8, 15.7) | 6.7  (3.7, 11.8) | 5.4  (2.9, 9.9) | 3.7  (1.9, 8.0) | 0.002 |  | 12.2  (6.8, 21.0) | 6.5  (2.9, 11.8) | 4.1  (2.2, 5.6) | 3.2  (2.0, 7.6) | <0.001 | |
| Percent with no health insurance, median (IQR) | 9.7  (7.0, 14.9) | 8.4  (5.7, 13.4) | 6.7  (4.7, 11.4) | 5.3  (2.6, 8.2) | <0.001 |  | 11.8  (8.7, 16.2) | 7.3  (5.1, 12.3) | 6.1  (4.5, 8.3) | 4.5  (2.3, 8.1) | <0.001 | |
|  |  |  |  |  |  |  |  |  |  |  |  | |
| Percent in Healthcare Industry, median (IQR) | 20.6  (17.4, 23.9) | 21.2  (19.6, 23.6) | 22.9  (21.7, 25.1) | 24.3  (19.7, 27.8) | 0.007 |  | 21.2  (17.3, 24.8) | 21.2  (18.5, 23.3) | 22.6  (21.1, 25.0) | 25.1  (20.1, 27.8) | 0.002 | |
| Percent in Service Industry, median (IQR) | 17.2  (14.3, 20.5) | 17.8  (14.3, 22.0) | 15.1  (12.1, 19.9) | 13.2  (9.1, 17.0) | <0.001 |  | 19.0  (14.6, 26.1) | 16.8  (13.7, 20.1) | 14.9  (12.4, 19.5) | 12.2  (9.1, 15.9) | <0.001 | |
| Percent Commuting via Public Transportation, median (IQR) | 0.1  (0.0, 1.2) | 0.4  (0.0, 2.0) | 0.4  (0.1, 2.8) | 0.7  (0.1, 2.2) | 0.096 |  | 0.3  (0.0, 5.5) | 0.2  (0.0, 2.0) | 0.4  (0.1, 1.4) | 0.5  (0.1, 1.6) | 0.59 | |
| Percent Working from Home, median (IQR) | 3.7  (2.1, 4.9) | 3.7  (2.7, 4.8) | 5.2  (3.8, 6.8) | 5.7  (3.6, 7.6) | <0.001 |  | 3.5  (2.2, 4.9) | 3.8  (2.7, 4.9) | 5.2  (4.0, 7.2) | 6.1  (3.7, 7.6) | <0.001 | |
|  |  |  |  |  |  |  |  |  |  |  |  | |
| Cases per 100,000 population, median (IQR) | 22,833  (20,914, 25,495) | 21,537  (19,506, 23,244) | 20,856  (17,005, 21,801) | 17,883  (15,841, 20,526) | <0.001 |  | 22,126  (19,825, 23,971) | 21,607  (19,476, 23,625) | 21,601  (18,472, 22,839) | 18,344  (15,741, 20,871) | <0.001 | |
| Deaths per 100,000 population, median (IQR) | 262  (173, 361) | 216  (165, 305) | 187  (122, 246) | 131  (60, 229) | <0.001 |  | 272  (199, 371) | 198  (123, 283) | 202  (136, 239) | 129  (59, 246) | <0.001 | |
| Vaccine Locations per 10,000 population, median (IQR) | 2.8  (1.6, 4.4) | 2.9  (2.0, 3.5) | 3.5  (2.1, 5.5) | 2.9  (2.3, 5.5) | 0.33 |  | 2.7  (1.6, 3.8) | 2.9  (2.0, 4.8) | 3.3  (2.1, 5.2) | 3.7  (2.3, 5.2) | 0.16 | |
|  |  |  |  |  |  |  |  |  |  |  |  | |
|  |  |  |  |  |  |  |  |  |  |  |  | |
| Overall SVI, median (IQR) | 50.3  (28.5, 69.2) | 32.9  (18.1, 48.2) | 31.7  (16.2, 46.7) | 23.8  (12.5, 44.5) | <0.001 |  | 53.5  (30.0, 76.7) | 39.7  (25.3, 49.2) | 23.5  (15.0, 34.1) | 23.1  (11.9, 44.4) | <0.001 | |
| Socioeconomic  theme, median  (IQR) | 51.5  (36.0, 69.2) | 40.0  (22.6, 53.6) | 35.5  (17.2, 53.2) | 25.3  (12.4, 49.2) | <0.001 |  | 56.0  (39.4, 79.3) | 44.6  (29.6, 60.4) | 23.1  (15.3, 35.3) | 22.2  (10.6, 48.0) | <0.001 | |
| Household  Composition  theme, median  (IQR) | 66.7  (50.0, 80.2) | 51.6  (34.2, 67.3) | 49.0  (32.7, 64.0) | 34.5  (17.3, 52.9) | <0.001 |  | 68.9  (49.4, 82.6) | 57.1  (37.8, 67.9) | 40.9  (30.2, 52.9) | 34.6  (21.9, 50.7) | <0.001 | |
| Minority  Status/Language  Theme, median  (IQR) | 21.4  (8.8, 54.5) | 26.7  (11.4, 49.8) | 32.0  (21.1, 51.6) | 31.5  (19.4, 46.2) | 0.23 |  | 22.3  (9.5, 56.1) | 22.9  (12.5, 52.8) | 28.1  (19.2, 44.1) | 31.2  (20.6, 42.7) | 0.82 | |
| Infrastructure  theme, median  (IQR) | 51.2  (36.5, 63.3) | 36.3  (26.1, 53.7) | 34.9  (21.5, 48.3) | 36.9  (20.7, 63.0) | 0.017 |  | 56.6  (35.0, 66.4) | 38.8  (26.1, 53.7) | 34.4  (18.0, 43.3) | 35.6  (20.3, 53.3) | <0.001 | |
|  |  |  |  |  |  |  |  |  |  |  |  | |
| Percent receiving at least one vaccine dose, median (IQR) | 43.3  (38.8, 49.6) | 50.0  (46.9, 56.9) | 57.1  (46.5, 60.2) | 61.6  (53.4, 68.9) | <0.001 |  | 16.3  (14.2, 20.2) | 23.6  (20.9, 26.5) | 29.3  (25.5, 31.7) | 35.3  (28.7, 39.7) | <0.001 | |
|  |  |  |  |  |  |  |  |  |  |  |  | |
| Percent Vaccinated at: |  |  |  |  |  |  |  |  |  |  |  | |
| Small Volume  Health Facility,  median (IQR) | 3.7  (2.5, 5.4) | 3.0  (2.4, 3.6) | 3.1  (2.6, 3.6) | 2.5  (2.1, 3.4) | 0.002 |  | 3.4  (2.3, 4.5) | 2.8  (2.1, 3.7) | 2.5  (2.0, 3.2) | 2.4  (2.0, 3.1) | 0.003 | |
| Medium Volume  Health Facility,  median (IQR) | 13.7  (9.3, 18.2) | 11.8  (9.5, 17.0) | 12.5  (10.0, 18.8) | 11.6  (8.3, 15.7) | 0.18 |  | 14.0  (9.8, 20.4) | 12.7  (9.4, 19.5) | 12.4  (10.5, 17.9) | 11.8  (10.1, 19.9) | 0.76 | |
| Large Volume  Health Facility,  median (IQR) | 13.8  (7.5, 25.8) | 22.6  (11.4, 27.7) | 22.5  (13.6, 28.0) | 25.5  (9.5, 33.2) | 0.001 |  | 20.7  (11.0, 37.7) | 28.9  (14.5, 35.5) | 33.2  (17.9, 39.6) | 34.9  (13.6, 41.9) | 0.013 | |
| Pharmacy,  median (IQR) | 42.7  (35.7, 47.1) | 40.4  (35.7, 46.7) | 36.9  (31.9, 43.1) | 33.0  (28.0, 45.0) | <0.001 |  | 27.1  (22.0, 35.3) | 28.7  (24.7, 33.0) | 25.8  (21.2, 33.1) | 23.7  (21.0, 34.6) | 0.24 | |
| Health  department,  median (IQR) | 17.4  (12.4, 23.2) | 16.9  (14.2, 18.9) | 17.6  (15.2, 19.4) | 17.9  (14.8, 21.5) | 0.39 |  | 19.6  (14.5, 26.3) | 21.4  (16.7, 24.0) | 20.8  (16.1, 25.1) | 17.4  (14.2, 23.4) | 0.18 | |
| Employer/school,  median (IQR) | 0.6  (0.0, 1.0) | 1.0  (0.6, 2.4) | 1.6  (1.0, 2.9) | 1.7  (0.0, 3.6) | <0.001 |  | 0.7  (0.0, 1.5) | 1.2  (0.5, 2.3) | 1.5  (0.9, 2.7) | 2.0  (0.0, 3.6) | 0.003 | |
| Other,  median (IQR) | 1.5  (0.9, 2.3) | 1.8  (1.4, 2.4) | 2.3  (1.9, 2.7) | 1.8  (0.5, 2.4) | 0.004 |  | 1.6  (0.6, 2.6) | 1.8  (1.2, 2.6) | 2.1  (1.4, 2.9) | 1.9  (0.0, 2.6) | 0.34 | |
|  |  |  |  |  |  |  |  |  |  |  |  | |
| Percent of Vaccinated Receiving: |  |  |  |  |  |  |  |  |  |  |  | |
| J&J,  median (IQR) | 7.2  (5.5, 8.8) | 6.6  (5.4, 7.9) | 6.7  (5.4, 7.7) | 6.7  (4.5, 8.9) | 0.74 |  | 6.6  (3.9, 8.1) | 6.4  (4.8, 7.8) | 4.6  (4.1, 6.8) | 4.4  (3.0, 7.1) | 0.033 | |
| Moderna,  median (IQR) | 60.0  (50.7, 66.7) | 64.6  (57.7, 68.2) | 65.1  (62.8, 69.0) | 65.4  (60.0, 70.7) | 0.001 |  | 54.9  (51.0, 64.7) | 59.8  (54.9, 65.8) | 65.0  (59.7, 67.9) | 65.5  (57.7, 71.2) | <0.001 | |
| Pfizer,  median (IQR) | 30.5  (26.2, 41.0) | 28.7  (25.8, 33.7) | 27.5  (25.1, 30.7) | 26.7  (23.2, 31.4) | 0.002 |  | 37.7  (29.9, 42.7) | 32.0  (27.7, 38.8) | 29.8  (26.7, 33.1) | 27.9  (24.4, 35.4) | <0.001 | |
|  |  |  |  |  |  |  |  |  |  |  |  | |
| Region |  |  |  |  |  |  |  |  |  |  |  | |
| Kansas City, n (%) | 32  (40.5%) | 28  (50.9%) | 16  (39.0%) | 35  (37.6%) | 0.44 |  | 28  (35.4%) | 30  (48.4%) | 21  (50.0%) | 28  (35.9%) | 0.2 | |
| St. Louis, n (%) | 47  (59.5%) | 27  (49.1%) | 25  (61.0%) | 58  (62.4%) |  |  | 51  (64.6%) | 32  (51.6%) | 21  (50.0%) | 50  (64.1%) |  |  |

*Notes*: Each quartile corresponds to successive segments of the Lorenz curve so that each quartile contains sufficient consecutive zip codes to account for 25% of diagnosed cases. Lorenz curve-based quartiles were generated by first sorting zip codes by their ratio of COVID-19 vaccinations to cases and splitting them such that each quartile accounted for 25% of the overall case number. Thus, the first quartile represents zip codes on the leftmost side of the curve (i.e., have the lowest ratio of COVID-19 vaccinations to cases) and the last quartile represents the zip codes on the rightmost side of the curve (i.e., have the highest ratio of COVID-19 vaccinations to cases). P-values were generated based on Kruskal-Wallis tests to assess differences between quartiles. Abbreviations: IQR=interquartile range; SVI=Social Vulnerability Index; J&J=Johnson and Johnson.
