# Supplementary material for: Quantifying inequities in COVID-19 vaccine distribution over time by social vulnerability, race and ethnicity, and location: A population-level analysis in St. Louis and Kansas City, Missouri
Source: PLoS Med. 2022 Aug 26;19(8):e1004048. doi: 10.1371/journal.pmed.1004048 (PMC9417193; doi:10.1371/journal.pmed.1004048)
Supplement: S6 Table — (DOCX) [file pmed.1004048.s014.docx]

| **S6 Table. Characteristics of Zip Codes by Quartile of Lorenz Curve – Number of COVID-19 Vaccinations relative to Deaths due to COVID-19** | | | | | | | | | | | |  |
| --- | --- | --- | --- | --- | --- | --- | --- | --- | --- | --- | --- | --- |
|  | **Primary Series** | | | | |  | **Booster** | | | | | |
|  | Lowest Quartile (n=45) | Second Quartile  (n=39) | Third Quartile  (n=36) | Highest Quartile  (n=148) | p-value |  | Lowest Quartile (n=48) | Second Quartile  (n=41) | Third Quartile  (n=36) | Highest Quartile  (n=136) | p-value | |
|  |  |  |  |  |  |  |  |  |  |  |  | |
| Percent of COVID-19 Vaccinations | 10.8 | 18.5 | 26.5 | 44.2 | <0.001 |  | 8.0 | 19.0 | 26.8 | 46.2 | <0.001 | |
| Percent of Deaths | 23.9 | 25.5 | 25.5 | 25.1 | <0.001 |  | 21.2 | 28.4 | 25.2 | 25.1 | <0.001 | |
|  |  |  |  |  |  |  |  |  |  |  |  | |
| Total Population, median (IQR) | 6,480  (475, 13,794) | 15,402  (4,683, 24,099) | 17,162  (10,218, 33,686) | 11,107  (3,022, 20,455) | <0.001 |  | 72,75  (565, 14,120) | 12,004  (4,775, 23,269) | 19,831  (9,968, 34,240) | 11,594  (2,249, 20,822) | <0.001 | |
| Percent Black, median (IQR) | 2.9  (0.1, 65.8) | 8.4  (0.9, 34.7) | 4.4  (1.8, 11.9) | 4.2  (0.5, 10.1) | 0.37 |  | 14.2  (0.3, 78.7) | 2.9  (0.8, 12.5) | 4.6  (2.2, 20.9) | 4.1  (0.5, 8.9) | 0.059 | |
| Zip Codes greater than 25% Black, n (%) | 16  (36%) | 12  (31%) | 5  (14%) | 15  (16%) | 0.018 |  | 21  (45%) | 8  (20%) | 7  (19%) | 12  (14%) | <0.001 | |
|  |  |  |  |  |  |  |  |  |  |  |  | |
| Percent Male, median (IQR) | 48.7  (46.9, 51.6) | 48.5  (46.8, 50.1) | 48.8  (48.0, 49.8) | 49.3  (48.0, 50.7) | 0.34 |  | 48.2  (45.9, 51.8) | 49.4  (47.4, 50.2) | 48.8  (48.2, 50.3) | 49.0  (47.5, 50.7) | 0.44 | |
| Median Age, median (IQR) | 38.6  (35.0, 43.6) | 38.5  (36.2, 43.2) | 39.0  (36.0, 40.8) | 38.3  (34.5, 41.9) | 0.59 |  | 38.1  (34.1, 40.9) | 38.5  (36.5, 43.1) | 38.5  (36.0, 40.8) | 39.0  (34.5, 42.4) | 0.54 | |
| Average Household Size, median (IQR) | 3.1  (2.9, 3.3) | 3.1  (3.0, 3.3) | 3.1  (3.0, 3.2) | 3.0  (2.9, 3.2) | 0.22 |  | 3.2  (2.9, 3.3) | 3.0  (3.0, 3.2) | 3.1  (2.9, 3.1) | 3.0  (2.8, 3.2) | 0.094 | |
|  |  |  |  |  |  |  |  |  |  |  |  | |
| Median Income ($), median (IQR) | 45,732  (31,200, 62,854) | 55,363  (45,663, 71,611) | 64,092  (50,288, 73,752) | 66,591  (48,105, 85,259) | <0.001 |  | 39,639  (24,826, 55,805) | 58,355  (47,589, 68,556) | 64,042  (47,612, 74,968) | 70,790  (54,004, 90,014) | <0.001 | |
| Percent below poverty line, median (IQR) | 9.4  (3.3, 18.5) | 7.8  (5.0, 14.1) | 6.8  (3.8, 12.8) | 4.3  (1.9, 9.8) | <0.001 |  | 12.3  (5.6, 23.8) | 7.6  (5.1, 12.2) | 6.3  (3.8, 12.8) | 3.7  (1.7, 8.3) | <0.001 | |
| Percent with no health insurance, median (IQR) | 11.5  (8.3, 15.7) | 8.7  (5.3, 13.4) | 7.1  (5.3, 10.9) | 6.0  (3.7, 8.7) | <0.001 |  | 13.4  (9.4, 16.7) | 8.4  (5.5, 11.4) | 7.1  (5.3, 10.9) | 5.5  (2.8, 8.1) | <0.001 | |
|  |  |  |  |  |  |  |  |  |  |  |  | |
| Percent in Healthcare Industry, median (IQR) | 22.5  (15.4, 26.2) | 23.0  (19.0, 25.2) | 22.7  (21.2, 24.9) | 21.7  (18.8, 25.3) | 0.65 |  | 22.3  (15.0, 26.5) | 22.9  (19.0, 25.1) | 22.5  (20.7, 24.2) | 21.9  (18.8, 25.6) | 0.92 | |
| Percent in Service Industry, median (IQR) | 17.5  (13.9, 28.7) | 18.0  (14.0, 22.0) | 16.0  (13.7, 20.0) | 14.6  (10.8, 18.5) | 0.005 |  | 19.9  (15.1, 29.6) | 17.7  (14.0, 20.0) | 17.3  (14.0, 20.4) | 13.9  (10.3, 16.9) | <0.001 | |
| Percent Commuting via Public Transportation, median (IQR) | 0.4  (0.0, 6.1) | 0.6  (0.0, 2.8) | 0.4  (0.1, 2.0) | 0.3  (0.0, 2.0) | 0.5 |  | 1.1  (0.0, 8.7) | 0.3  (0.0, 1.3) | 0.4  (0.2, 2.3) | 0.3  (0.0, 1.2) | 0.022 | |
| Percent Working from Home, median (IQR) | 3.5  (2.1, 5.9) | 3.9  (2.4, 4.7) | 4.4  (2.8, 5.4) | 5.2  (3.5, 7.6) | 0.005 |  | 3.5  (2.1, 4.9) | 3.4  (2.6, 4.9) | 4.2  (2.8, 4.9) | 5.6  (3.7, 7.6) | <0.001 | |
|  |  |  |  |  |  |  |  |  |  |  |  | |
| Cases per 100,000 population, median (IQR) | 21,274  (18,920, 23,742) | 22,092  (20,490, 23,951) | 21,444  (19,875, 23,001) | 19,922  (16,614, 22,642) | 0.003 |  | 21,634  (19,292, 23,945) | 22,050  (20,227, 23,777) | 21,281  (19,561, 22,770) | 19,938  (16,650, 22,523) | 0.009 | |
| Deaths per 100,000 population, median (IQR) | 424  (359, 609) | 279  (242, 315) | 225  (194, 243) | 113  (51, 151) | <0.001 |  | 385  (340, 561) | 268  (214, 314) | 217  (175, 239) | 107  (49, 152) | <0.001 | |
| Vaccine Locations per 10,000 population, median (IQR) | 2.7  (1.8, 6.0) | 3.4  (2.3, 4.8) | 3.0  (1.9, 4.1) | 3.0  (2.0, 4.7) | 0.8 |  | 2.7  (1.7, 4.5) | 3.3  (2.3, 4.5) | 2.9  (1.8, 5.0) | 3.2  (2.2, 4.7) | 0.59 | |
|  |  |  |  |  |  |  |  |  |  |  |  | |
|  |  |  |  |  |  |  |  |  |  |  |  | |
| Overall SVI, median (IQR) | 50.3  (23.8, 74.6) | 47.1  (25.3, 66.9) | 38.6  (24.1, 51.1) | 30.0  (12.9, 44.5) | <0.001 |  | 62.2  (31.5, 82.5) | 39.2  (25.3, 55.1) | 41.3  (24.1, 49.9) | 25.2  (12.5, 43.0) | <0.001 | |
| Socioeconomic  theme, median  (IQR) | 51.3  (34.5, 78.2) | 49.2  (26.0, 69.3) | 43.4  (23.3, 56.1) | 30.6  (15.3, 51.3) | <0.001 |  | 65.7  (39.8, 84.6) | 46.7  (26.0, 60.2) | 42.2  (22.8, 53.2) | 27.4  (13.6, 49.8) | <0.001 | |
| Household  Composition  theme, median  (IQR) | 68.9  (50.8, 83.3) | 66.7  (44.8, 75.3) | 52.4  (41.0, 65.7) | 38.8  (22.1, 56.7) | <0.001 |  | 79.0  (56.6, 84.4) | 63.3  (35.7, 71.6) | 47.9  (40.7, 60.7) | 38.1  (22.2, 55.9) | <0.001 | |
| Minority  Status/Language  Theme, median  (IQR) | 22.6  (9.2, 54.4) | 40.3  (11.7, 55.6) | 27.3  (17.8, 50.9) | 27.6  (18.5, 46.2) | 0.8 |  | 44.1  (10.3, 58.2) | 21.8  (9.5, 48.7) | 33.5  (20.8, 52.1) | 26.3  (18.6, 41.5) | 0.23 | |
| Infrastructure  theme, median  (IQR) | 48.6  (35.0, 61.6) | 45.6  (32.2, 58.0) | 45.8  (33.0, 56.3) | 34.7  (20.7, 62.2) | 0.21 |  | 52.6  (38.7, 63.3) | 40.3  (32.2, 56.8) | 41.2  (28.8, 54.9) | 34.4  (18.6, 54.6) | 0.01 | |
|  |  |  |  |  |  |  |  |  |  |  |  | |
| Percent receiving at least one vaccine dose, median (IQR) | 44.5  (39.2, 51.7) | 48.8  (41.7, 53.7) | 53.1  (48.8, 57.6) | 55.9  (47.4, 63.0) | <0.001 |  | 16.4  (14.2, 21.5) | 21.4  (16.6, 26.4) | 24.9  (22.1, 29.0) | 29.6  (22.1, 35.6) | <0.001 | |
|  |  |  |  |  |  |  |  |  |  |  |  | |
| Percent Vaccinated at: |  |  |  |  |  |  |  |  |  |  |  | |
| Small Volume  Health Facility,  median (IQR) | 3.7  (2.9, 4.7) | 3.2  (2.6, 4.4) | 2.8  (2.6, 3.5) | 2.7  (1.9, 3.7) | <0.001 |  | 3.5  (2.4, 4.5) | 3.0  (2.3, 3.7) | 2.7  (2.4, 3.4) | 2.4  (0.0, 3.3) | <0.001 | |
| Medium Volume  Health Facility,  median (IQR) | 11.4  (9.3, 13.2) | 14.1  (10.6, 17.8) | 12.3  (10.6, 17.2) | 11.9  (7.8, 18.7) | 0.081 |  | 12.1  (9.5, 17.9) | 13.1  (10.5, 18.7) | 13.9  (11.6, 21.4) | 12.3  (9.0, 20.0) | 0.16 | |
| Large Volume  Health Facility,  median (IQR) | 22.0  (11.5, 27.7) | 15.2  (9.6, 28.6) | 26.2  (12.1, 29.5) | 20.9  (8.8, 28.9) | 0.59 |  | 29.3  (15.7, 38.1) | 29.1  (13.9, 38.9) | 29.3  (12.4, 38.0) | 29.0  (12.8, 39.2) | 0.99 | |
| Pharmacy,  median (IQR) | 41.7  (36.7, 48.2) | 39.9  (32.6, 44.9) | 39.1  (34.8, 45.7) | 35.7  (29.0, 45.9) | 0.015 |  | 28.2  (23.9, 32.6) | 27.0  (22.1, 31.9) | 27.4  (22.6, 32.3) | 25.0  (20.9, 34.5) | 0.57 | |
| Health  department,  median (IQR) | 14.9  (13.4, 17.8) | 19.0  (15.2, 22.2) | 17.1  (15.2, 19.1) | 18.0  (14.2, 21.6) | 0.014 |  | 18.3  (14.6, 23.7) | 21.2  (16.9, 25.2) | 22.5  (17.5, 24.1) | 19.1  (12.6, 24.3) | 0.11 | |
| Employer/school,  median (IQR) | 0.9  (0.4, 3.5) | 0.9  (0.6, 1.5) | 1.0  (0.7, 2.3) | 1.2  (0.0, 2.6) | 0.66 |  | 0.9  (0.3, 3.4) | 1.1  (0.7, 2.2) | 1.3  (0.8, 2.1) | 1.3  (0.0, 2.9) | 0.6 | |
| Other,  median (IQR) | 2.0  (1.5, 3.5) | 1.8  (1.4, 2.8) | 1.9  (1.4, 2.4) | 1.6  (0.0, 2.3) | 0.018 |  | 2.1  (1.4, 3.3) | 1.7  (1.2, 2.5) | 2.0  (1.7, 2.6) | 1.6  (0.0, 2.5) | 0.008 | |
|  |  |  |  |  |  |  |  |  |  |  |  | |
| Percent of Vaccinated Receiving: |  |  |  |  |  |  |  |  |  |  |  | |
| J&J,  median (IQR) | 7.0  (4.8, 7.8) | 6.9  (5.5, 8.5) | 6.9  (5.5, 8.0) | 6.9  (4.6, 9.0) | 0.95 |  | 6.7  (4.8, 8.1) | 5.6  (4.4, 8.1) | 6.4  (4.2, 7.7) | 4.8  (2.8, 7.1) | 0.008 | |
| Moderna,  median (IQR) | 62.2  (56.6, 66.0) | 63.8  (54.7, 68.4) | 65.3  (59.7, 67.8) | 64.6  (58.1, 70.2) | 0.099 |  | 56.5  (51.7, 65.1) | 57.4  (53.8, 66.8) | 62.3  (58.7, 65.7) | 64.1  (55.7, 70.0) | 0.003 | |
| Pfizer,  median (IQR) | 30.0  (27.1, 35.6) | 29.6  (25.5, 38.9) | 27.8  (25.5, 32.7) | 27.1  (23.0, 33.1) | 0.009 |  | 36.5  (30.2, 40.9) | 35.3  (27.6, 40.1) | 30.7  (28.6, 33.7) | 28.7  (24.9, 37.5) | 0.003 | |
|  |  |  |  |  |  |  |  |  |  |  |  | |
| Region |  |  |  |  |  |  |  |  |  |  |  | |
| Kansas City, n (%) | 18  (40.0%) | 17  (43.6%) | 16  (44.4%) | 60  (40.5%) | 0.96 |  | 22  (45.8%) | 13  (31.7%) | 18  (50.0%) | 54  (39.7%) | 0.36 | |
| St. Louis, n (%) | 27  (60.0%) | 22  (56.4%) | 20  (55.6%) | 88  (59.5%) |  |  | 26  (54.2%) | 28  (68.3%) | 18  (50.0%) | 82  (60.3%) |  |  |

*Notes*: Each quartile corresponds to successive segments of the Lorenz curve so that each quartile contains sufficient consecutive zip codes to account for 25% of COVID-19 deaths. Lorenz curve-based quartiles were generated by first sorting zip codes by their ratio of COVID-19 vaccinations to COVID-19 deaths and splitting them such that each quartile accounted for 25% of the overall number of deaths. Thus, the first quartile represents zip codes on the leftmost side of the curve (i.e., have the lowest ratio of COVID-19 vaccinations to deaths) and the last quartile represents the zip codes on the rightmost side of the curve (i.e., have the highest ratio of COVID-19 vaccinations to deaths). P-values were generated based on Kruskal-Wallis tests to assess differences between quartiles. Abbreviations: IQR=interquartile range; SVI=Social Vulnerability Index; J&J=Johnson and Johnson
