# Supplementary material for: Quantifying inequities in COVID-19 vaccine distribution over time by social vulnerability, race and ethnicity, and location: A population-level analysis in St. Louis and Kansas City, Missouri
Source: PLoS Med. 2022 Aug 26;19(8):e1004048. doi: 10.1371/journal.pmed.1004048 (PMC9417193; doi:10.1371/journal.pmed.1004048)
Supplement: S7 Table — (DOCX) [file pmed.1004048.s015.docx]

| **S7 Table. Characteristics of Zip Codes by Quartile of Lorenz Curve – Number of COVID-19 Vaccinations relative to Total SVI** | | | | | | | | | | | | | | | | | | |  |
| --- | --- | --- | --- | --- | --- | --- | --- | --- | --- | --- | --- | --- | --- | --- | --- | --- | --- | --- | --- |
|  | **Primary Series** | | | | |  | | **Booster** | | | | | | | | | |  |  |
|  | Lowest Quartile (n=28) | Second Quartile  (n=42) | Third Quartile  (n=50) | Highest Quartile  (n=91) | p-value |  | | Lowest Quartile (n=29) | | Second Quartile  (n=45) | | Third Quartile  (n=50) | | Highest Quartile  (n=87) | | p-value | |  |  |
|  |  |  |  |  |  |  | |  | |  | |  | |  | |  | |  |  |
| Percent of COVID-19 Vaccinations | 8.8 | 13.0 | 22.1 | 56.1 | <0.001 |  | | 6.1 | | 12.1 | | 20.5 | | 61.3 | | <0.001 | |  |  |
| Percent of Total SVI | 24.4 | 24.6 | 25.7 | 25.3 | <0.001 |  | | 23.7 | | 26.2 | | 24.7 | | 25.4 | | <0.001 | |  |  |
|  |  |  |  |  |  |  | |  | |  | |  | |  | |  | |  |  |
| Total Population, median (IQR) | 10,986  (6,584, 17,380) | 10,808  (4,313, 19,310) | 13,216  (2,381, 20,442) | 15,450  (3,038, 26,768) | 0.52 |  | | 10,469  (5,894, 16,504) | | 10,733  (3,501, 17,531) | | 10,898  (1,870, 22,133) | | 16,323  (5,014, 26,768) | | 0.17 | |  |  |
| Percent Black, median (IQR) | 63.0  (17.4, 89.2) | 4.4  (0.9, 34.7) | 3.4  (0.3, 21.5) | 3.1  (0.5, 6.8) | <0.001 |  | | 77.6  (24.8, 89.0) | | 4.4  (0.9, 32.5) | | 2.8  (0.3, 11.9) | | 3.3  (0.5, 7.0) | | <0.001 | |  |  |
| Zip Codes greater than 25% Black, n (%) | 19  (68%) | 14  (33%) | 11  (22%) | 4  (4%) | <0.001 |  | | 21  (72%) | | 14  (31%) | | 9  (18%) | | 4  (5%) | | <0.001 | |  |  |
|  |  |  |  |  |  |  | |  | |  | |  | |  | |  | |  |  |
| Percent Male, median (IQR) | 47.1  (44.5, 48.8) | 48.4  (47.1, 50.1) | 49.7  (48.3, 51.5) | 48.9  (48.2, 50.5) | <0.001 |  | | 46.9  (44.6, 48.3) | | 48.8  (47.6, 50.6) | | 49.7  (48.4, 51.5) | | 48.8  (48.1, 50.2) | | <0.001 | |  |  |
| Median Age, median (IQR) | 35.0  (33.0, 39.0) | 37.7  (35.0, 40.2) | 38.9  (35.7, 42.5) | 39.8  (36.4, 44.0) | <0.001 |  | | 34.5  (32.9, 38.9) | | 37.8  (35.0, 40.2) | | 39.2  (36.7, 44.0) | | 39.6  (35.7, 43.4) | | <0.001 | |  |  |
| Average Household Size, median (IQR) | 3.3  (3.2, 3.5) | 3.1  (3.0, 3.2) | 3.0  (2.9, 3.1) | 3.0  (2.9, 3.1) | <0.001 |  | | 3.3  (3.2, 3.4) | | 3.1  (3.0, 3.2) | | 3.0  (2.8, 3.1) | | 3.0  (2.9, 3.2) | | <0.001 | |  |  |
|  |  |  |  |  |  |  | |  | |  | |  | |  | |  | |  |  |
| Median Income ($), median (IQR) | 30,450  (24,186, 38,274) | 47,412  (43,511, 55,911) | 58,692  (48,105, 66,771) | 76,935  (63,896, 98,202) | <0.001 |  | | 30,177  (23,951, 38,682) | | 47,324  (41,875, 55,805) | | 60,216  (49,352, 67,250) | | 77,656  (64,097, 99,025) | | <0.001 | |  |  |
| Percent below poverty line, median (IQR) | 26.6  (16.6, 31.2) | 12.1  (8.9, 15.2) | 6.9  (5.3, 10.5) | 3.0  (1.6, 5.3) | <0.001 |  | | 26.4  (19.6, 31.3) | | 12.2  (8.9, 15.2) | | 6.8  (5.3, 10.1) | | 3.0  (1.6, 5.2) | | <0.001 | |  |  |
| Percent with no health insurance, median (IQR) | 16.4  (14.1, 23.0) | 10.1  (7.6, 14.1) | 8.2  (6.1, 11.5) | 4.9  (2.8, 6.9) | <0.001 |  | | 16.7  (14.7, 23.0) | | 10.8  (7.7, 14.1) | | 8.1  (6.1, 11.2) | | 4.5  (2.7, 6.6) | | <0.001 | |  |  |
|  |  |  |  |  |  |  | |  | |  | |  | |  | |  | |  |  |
| Percent in Healthcare Industry, median (IQR) | 24.3  (17.8, 29.7) | 20.8  (18.5, 23.2) | 22.3  (19.6, 24.7) | 22.7  (19.5, 25.6) | 0.11 |  | | 25.1  (19.0, 29.3) | | 20.8  (18.6, 23.2) | | 21.2  (16.9, 24.1) | | 22.9  (19.8, 26.2) | | 0.011 | |  |  |
| Percent in Service Industry, median (IQR) | 28.5  (22.0, 31.3) | 19.0  (14.6, 20.8) | 17.2  (14.4, 19.5) | 13.2  (9.1, 15.4) | <0.001 |  | | 28.8  (22.2, 31.5) | | 19.2  (15.1, 21.3) | | 16.3  (14.0, 19.3) | | 12.8  (9.1, 15.3) | | <0.001 | |  |  |
| Percent Commuting via Public Transportation, median (IQR) | 8.1  (2.0, 15.0) | 0.3  (0.0, 2.0) | 0.4  (0.0, 1.9) | 0.2  (0.0, 0.7) | <0.001 |  | | 7.4  (2.9, 14.7) | | 0.3  (0.0, 2.1) | | 0.2  (0.0, 1.0) | | 0.3  (0.0, 0.8) | | <0.001 | |  |  |
| Percent Working from Home, median (IQR) | 2.8  (2.1, 4.6) | 3.1  (2.4, 4.5) | 4.0  (3.2, 5.4) | 6.0  (4.0, 7.6) | <0.001 |  | | 3.0  (2.1, 4.5) | | 3.4  (2.7, 4.7) | | 3.7  (2.8, 4.7) | | 6.1  (4.2, 7.6) | | <0.001 | |  |  |
|  |  |  |  |  |  |  | |  | |  | |  | |  | |  | |  |  |
| Cases per 100,000 population, median (IQR) | 19,499  (16,004, 21,017) | 21,276  (17,779, 22,833) | 20,983  (17,092, 22,781) | 21,638  (19,506, 24,100) | 0.005 |  | | 19,866  (17,006, 21,619) | | 20,125  (16,580, 22,708) | | 21,363  (19,107, 23,463) | | 21,638  (19,506, 23,990) | | 0.017 | |  |  |
| Deaths per 100,000 population, median (IQR) | 272  (201, 349) | 230  (162, 342) | 196  (122, 279) | 186  (100, 259) | 0.058 |  | | 277  (216, 353) | | 228  (130, 340) | | 191  (122, 279) | | 186  (100, 258) | | 0.011 | |  |  |
| Vaccine Locations per 10,000 population, median (IQR) | 2.1  (1.6, 2.9) | 2.5  (1.8, 3.8) | 3.7  (2.7, 4.8) | 3.2  (2.3, 5.0) | 0.009 |  | | 2.1  (1.5, 3.0) | | 2.7  (1.9, 3.8) | | 3.7  (2.7, 4.8) | | 3.2  (2.3, 5.0) | | 0.026 | |  |  |
|  |  |  |  |  |  |  | |  | |  | |  | |  | |  | |  |  |
|  |  |  |  |  |  |  | |  | |  | |  | |  | |  | |  |  |
| Overall SVI, median (IQR) | 84.5  (75.7, 87.7) | 54.7  (46.9, 64.1) | 41.3  (32.7, 47.1) | 16.4  (11.9, 23.8) | <0.001 |  | | 83.6  (74.6, 87.6) | | 53.8  (45.6, 63.0) | | 39.0  (30.3, 47.1) | | 16.4  (11.0, 23.8) | | <0.001 | |  |  |
| Socioeconomic  theme, median  (IQR) | 84.1  (78.7, 92.1) | 58.9  (49.2, 67.8) | 43.4  (35.1, 50.1) | 18.6  (10.8, 31.8) | <0.001 |  | | 85.8  (78.4, 91.9) | | 58.6  (48.5, 67.3) | | 42.7  (36.0, 49.9) | | 18.4  (10.7, 27.5) | | <0.001 | |  |  |
| Household  Composition  theme, median  (IQR) | 84.7  (79.2, 89.3) | 68.8  (55.6, 75.3) | 55.6  (44.7, 64.2) | 35.0  (24.1, 46.3) | <0.001 |  | | 84.5  (79.5, 88.4) | | 67.7  (54.9, 72.7) | | 55.6  (45.2, 66.1) | | 34.5  (24.1, 46.0) | | <0.001 | |  |  |
| Minority  Status/Language  Theme, median  (IQR) | 56.3  (47.3, 70.6) | 30.4  (9.2, 56.9) | 22.9  (13.8, 48.7) | 23.9  (14.9, 32.3) | <0.001 |  | | 56.1  (48.9, 65.4) | | 27.6  (9.5, 56.9) | | 21.4  (11.4, 47.2) | | 24.2  (14.9, 32.9) | | <0.001 | |  |  |
| Infrastructure  theme, median  (IQR) | 65.7  (55.5, 74.5) | 56.6  (42.4, 66.1) | 44.0  (32.3, 53.2) | 23.5  (16.1, 35.1) | <0.001 |  | | 62.2  (52.0, 71.2) | | 56.6  (42.4, 67.9) | | 41.7  (29.2, 52.1) | | 23.6  (16.3, 36.0) | | <0.001 | |  |  |
|  |  |  |  |  |  |  | |  | |  | |  | |  | |  | |  |  |
| Percent receiving at least one vaccine dose, median (IQR) | 43.6  (40.2, 46.1) | 43.1  (38.8, 49.1) | 51.8  (47.3, 56.6) | 60.1  (53.2, 67.5) | <0.001 |  | | 14.7  (13.1, 15.6) | | 18.7  (15.7, 20.7) | | 23.7  (21.4, 26.9) | | 32.3  (27.5, 38.5) | | <0.001 | |  |  |
|  |  |  |  |  |  |  | |  | |  | |  | |  | |  | |  |  |
| Percent Vaccinated at: |  |  |  |  |  |  | |  | |  | |  | |  | |  | |  |  |
| Small Volume  Health Facility,  median (IQR) | 3.8  (3.2, 4.3) | 3.4  (3.0, 4.9) | 2.8  (2.5, 3.7) | 2.6  (2.4, 3.4) | <0.001 |  | | 3.1  (2.4, 3.9) | | 3.3  (2.4, 3.9) | | 2.7  (2.3, 3.7) | | 2.6  (2.2, 3.3) | | 0.044 | |  |  |
| Medium Volume  Health Facility,  median (IQR) | 12.4  (9.9, 15.5) | 14.2  (9.2, 17.4) | 11.7  (9.6, 16.6) | 12.2  (10.2, 17.9) | 0.85 |  | | 13.3  (9.9, 17.6) | | 17.0  (9.9, 21.0) | | 11.9  (10.5, 18.0) | | 12.7  (10.8, 19.9) | | 0.46 | |  |  |
| Large Volume  Health Facility,  median (IQR) | 18.2  (11.2, 24.1) | 13.8  (8.7, 24.0) | 21.6  (13.6, 27.7) | 27.3  (14.8, 32.7) | <0.001 |  | | 24.1  (16.9, 36.6) | | 20.2  (11.7, 34.7) | | 28.6  (14.7, 38.2) | | 35.5  (21.2, 40.6) | | 0.003 | |  |  |
| Pharmacy,  median (IQR) | 39.4  (36.1, 45.7) | 42.9  (35.9, 48.2) | 39.1  (32.2, 44.7) | 36.5  (30.6, 45.5) | 0.024 |  | | 25.1  (23.2, 30.8) | | 30.0  (23.8, 35.2) | | 27.1  (23.8, 32.5) | | 25.0  (22.4, 32.8) | | 0.32 | |  |  |
| Health  department,  median (IQR) | 16.6  (14.5, 20.6) | 19.9  (15.2, 24.0) | 17.8  (15.2, 21.5) | 16.8  (14.8, 19.3) | 0.03 |  | | 20.9  (18.3, 26.3) | | 23.4  (17.3, 26.7) | | 22.0  (17.2, 25.4) | | 18.7  (14.9, 23.0) | | 0.001 | |  |  |
| Employer/school,  median (IQR) | 3.2  (0.5, 4.3) | 0.8  (0.5, 1.1) | 1.0  (0.7, 2.6) | 1.6  (0.7, 2.4) | 0.008 |  | | 2.7  (0.8, 4.4) | | 0.8  (0.6, 1.4) | | 1.1  (0.7, 2.6) | | 1.7  (0.8, 2.6) | | 0.01 | |  |  |
| Other,  median (IQR) | 2.9  (2.1, 3.7) | 1.8  (1.4, 2.3) | 1.6  (1.3, 2.2) | 2.0  (1.5, 2.6) | <0.001 |  | | 2.6  (1.9, 3.7) | | 1.8  (1.2, 2.5) | | 1.7  (1.3, 2.4) | | 2.0  (1.6, 2.6) | | 0.013 | |  |  |
|  |  |  |  |  |  |  | |  | |  | |  | |  | |  | |  |  |
| Percent of Vaccinated Receiving: |  |  |  |  |  |  | |  | |  | |  | |  | |  | |  |  |
| J&J,  median (IQR) | 7.5  (7.1, 9.1) | 7.2  (5.8, 8.6) | 7.2  (5.7, 9.0) | 5.6  (4.8, 7.5) | <0.001 |  | | 7.6  (6.9, 9.6) | | 6.5  (4.6, 8.3) | | 6.7  (5.0, 8.1) | | 4.7  (3.9, 6.7) | | <0.001 | |  |  |
| Moderna,  median (IQR) | 62.4  (57.7, 65.8) | 60.7  (49.9, 64.9) | 61.9  (56.6, 64.6) | 67.9  (64.3, 69.7) | <0.001 |  | | 57.3  (52.6, 61.4) | | 57.0  (51.0, 62.4) | | 58.9  (55.5, 64.5) | | 66.6  (62.7, 69.9) | | <0.001 | |  |  |
| Pfizer,  median (IQR) | 29.6  (26.0, 33.6) | 31.1  (26.9, 42.8) | 30.5  (26.7, 34.2) | 26.2  (23.5, 29.5) | <0.001 |  | | 34.5  (31.2, 39.2) | | 35.3  (30.5, 43.6) | | 33.2  (27.9, 39.0) | | 27.8  (25.2, 31.5) | | <0.001 | |  |  |
|  |  |  |  |  |  |  | |  | |  | |  | |  | |  | |  |  |
| Region |  |  |  |  |  |  | |  | |  | |  | |  | |  | |  |  |
| Kansas City, n (%) | 11  (39.3%) | 18  (42.9%) | 22  (44.0%) | 37  (40.7%) | 0.97 |  | | 12  (41.4%) | | 19  (42.2%) | | 23  (46.0%) | | 34  (39.1%) | | 0.89 | |  |  |
| St. Louis, n (%) | 17  (60.7%) | 24  (57.1%) | 28  (56.0%) | 54  (59.3%) |  | |  | | 17  (58.6%) | | 26  (57.8%) | | 27  (54.0%) | | 53  (60.9%) | |  | | |

*Notes*: Each quartile corresponds to successive segments of the Lorenz curve so that each quartile contains sufficient consecutive zip codes to account for 25% of population-level social vulnerability. Lorenz curve-based quartiles were generated by first sorting zip codes by their ratio of COVID-19 vaccinations to total social vulnerability and splitting them such that each quartile accounted for 25% of the total social vulnerability. Thus, the first quartile represents zip codes on the leftmost side of the curve (i.e., have the lowest ratio of COVID-19 vaccinations to total social vulnerability) and the last quartile represents the zip codes on the rightmost side of the curve (i.e., have the highest ratio of COVID-19 vaccinations to total social vulnerability). P-values were generated based on Kruskal-Wallis tests to assess differences between quartiles. Abbreviations: IQR=interquartile range; SVI=Social Vulnerability Index; J&J=Johnson and Johnson.
