# Supplementary material for: Quantifying inequities in COVID-19 vaccine distribution over time by social vulnerability, race and ethnicity, and location: A population-level analysis in St. Louis and Kansas City, Missouri
Source: PLoS Med. 2022 Aug 26;19(8):e1004048. doi: 10.1371/journal.pmed.1004048 (PMC9417193; doi:10.1371/journal.pmed.1004048)
Supplement: Attachment — Submitted filename: PLOS Med Response to Reviewers.docx [file pmed.1004048.s016.docx]

***RESPONSE TO REVIEWERS***

**Editorial Comments:**
Please adapt the competing interest statement (submission form) to include "EHG is a member of PLOS Medicine's Editorial Board" or similar.

***We have added this statement to the submission form. We apologize for the accidental omission in the original submission.***

We ask you to restructure the title to better match journal style and suggest: "Quantifying disparities in COVID-19 vaccine distribution by social vulnerability, ethnicity, location, and time: A population-level analysis".

Please also state where the study was done in the title.

***We have changed the title to “*Quantifying Inequities in COVID-19 Vaccine Distribution Over Time by social vulnerability, race and ethnicity, and location: A Population-Level Analysis in St. Louis and Kansas City, Missouri”**

Please add a new final sentence to the "Methods and findings" subsection of your abstract, which should begin "Study limitations include ..." or similar and should quote 2-3 of the study's main limitations.

***We have included the following sentence at the end of the abstract.***

***“Study limitations include inherent limitations in vaccine registry dataset such as missing and misclassified race/ethnicity and zip code variables and potential changes in zip code population sizes since census enumeration.***”

After the abstract, please add a new and accessible "Author summary" section in non-identical prose. You may find it helpful to consult one or two recent research papers published in PLOS Medicine to get a sense of the preferred style.

***We have now included an Author Summary after the abstract.***

The sentence at line 99 beginning "The novel application ..." is really an item of discussion, and so should be removed or relocated to the Discussion.

***Thank you for this comment. If acceptable to the editors, we would prefer to preserve this sentence in the introduction with some changes. We believe this sentence helps to frame our analysis by highlighting the potential importance and utility of the specific approach that we have taken. In response, we have reframed the sentence to highlight the “potential utility” of this approach rather than stating that it does (which we agree would be more appropriate in the discussion).***

***“The novel application of this methodology—which we previously used to characterize COVID-19 testing disparities [4]—has potential to yield deeper insights into the progress made towards vaccine equity in these regions that may then better inform health policy solutions to address remaining gaps.”***

Early in the Methods section (main text), please state whether the study had a protocol or prespecified analysis plan, and if so attach the relevant document as a supplementary file, referred to in the text.

Please highlight any non-prespecified analyses.

***We have included the following sentence in the beginning of the Methods section.***

***“The research in this paper was not pre-specified and consists of secondary analysis of preexisting de-identified data.”***

We suggest substituting "ethnicity" for "race" throughout.

***Thank you for this comment. In response to reviewer comments, we expanded our reporting to Black, White, Hispanic, and Asian populations. These categorizations combine elements of race (i.e., socially constructed categorizations based on phenotypic appearance, which are highly associated with experiencing systemic racism) and ethnicity (i.e., groupings based on shared family and cultural history traditions). Thus, we have changed the manuscript to use race and ethnicity throughout as opposed to just one.***

Please remove the information on funding and competing interests from the end of the main text. In the event of publication, this information will appear in the article metadata via entries in the submission form, and does not need to be duplicated.

***We have removed these sections and have included the information in the submission form.***

In the reference list, please use the journal name abbreviation "PLoS ONE".

***We have corrected the journal abbreviations***

Noting reference 4 and others, please ensure that all references have full access details.

***We apologize for the errors. We have corrected the citations.***

Please add a completed checklist for the most appropriate reporting guideline, e.g., STROBE, as an attachment, labelled "S1_STROBE_Checklist" or similar and referred to as such in the Methods section (main text).

In the checklist, please ensure that individual items are referred to by section (e.g., "Methods") and paragraph number, not by line or page numbers as these generally change in the event of publication.

***We have added a STROBE checklist as a supplemental appendix and also mention this is the Ethics statement at the beginning of the Methods section.***

**Academic Editor Comments:**

-       Equity is a very important, but often misunderstood concept – would it be possible to give the definition of equity that you use (and maybe also to distinguish from equality)?

**Thank you for the opportunity to clarify. We have added the following sentence to the second paragraph in the introduction.**

***“Whereas equality simply refers to provision of equal resources to every individual regardless of need, equitable approaches acknowledge that individuals will have different risks, needs, or opportunities and that access to or distribution of resources needs to take these differences into account.”***

-       Overlap with previous publications. You cite a previous study using the same methods in the introduction (line 100, ref 4, DOI: 10.1093/cid/ciaa1848) and you have a research letter on COVID-19 testing (<https://jamanetwork.com/journals/jamanetworkopen/articlepdf/2774715/mody_2021_ld_200197_1609254555.33582.pdf>). There is a fair bit of overlap in wording:

o       Introduction and Methods – particularly ref 4, the last para of the introduction and the headings and wording of the sections ‘Study Setting and Data’ and ‘Analyses’ use much of the same wording, replacing ‘testing’ with ‘vaccination’. Please cite these studies in the Methods, saying that you describe the same strategy as for ref 4 and apply it to vaccination.

***We include the following sentence in the methods to make clear that these methods are adapted from those that we had previously used.***

***“We adapted methods that we had previously used to assess disparities related to COVID-19 testing and extend them to COVID-19 vaccination [4].”***

o       Results, discussion, conclusion – there are chunks of repeated text in the results, showing similar patterns for vaccination as for testing. In the Discussion, you don’t mention the previous paper at all, but you draw the same conclusion in both papers – about the need for proactive public health strategies. It would make sense to discuss your findings about inequities in both vaccination and testing.

***Thank you for this comment. We agree with the academic editor that the parallel findings with our prior work examining COVID-19 testing disparities is noteworthy. They help to reinforce the systemic nature of these disparities given that they are repeatedly demonstrated. We include the following in the discussion to highlight these parallel findings.***

***“These patterns seen in both the primary vaccine series and booster rollout were also mirrored in prior research from our group examining disparities in COVID-19 testing, and their origins can be traced back to many of the same root causes [4]. Ultimately, the repeated reliance on systems with a history of providing lower access to certain segments of the population is representative of how structural inequities also became embedded in vaccine rollout from its onset and serves as a precautionary tale, albeit one that has been told too many times before.”***

-       Methods, lines 110-113: the study is done in 7 counties in St. Louis and 4 in Kansas City regions – can you say why you choose these counties? How many are there in total in each region?

***Thank you for the opportunity to clarify. These counties make up the wider metropolitan area for these two cities (which are the largest two cities in Missouri). Thus, the purpose was to include the full metropolitan area rather than just the city itself. No Missouri counties in the metropolitan areas were excluded, although it is important to note that we did not have access to data from counties in Illinois or Kansas (some of the metropolitan areas also extend into these states). We have rephrased the sentence to the below to be more clear.***

***“We sought to assess disparities in COVID-19 vaccination across the 7 counties in the St. Louis region (St. Louis City, St. Louis County, St. Charles, Jefferson, Franklin, Lincoln, and Warren; total population 2,095,978: 19.2% Black, 73.1% White, 3.0% Hispanic, 3.2% Asian) and the 4 counties in the Kansas City region (Jackson, Clay, Cass, and Platte; total population 1,121,224: 16.8% Black, 73.2% White, 8.2% Hispanic, 2.0% Asian). These counties make up the broader metropolitan area located within Missouri for these two cities.”***

-       Restriction to people identified as Black and White – please explain in the Methods why you don’t mention (except in tables) or conduct analyses for people from any other group – there are hundreds of thousands of people in these groups in your study populations. Omission of numerically large groups of people is only mentioned as a limitation (line 361).

***Thank you for this comment. This is an important point and, in response to Reviewer #1’s comments as well, we do now include Hispanic and Asian ethnicity as well in our analyses. Initially, we had intended to conduct analyses that were race-specific at the zip-code level, and we were concerned about small numbers in individual-level zip codes. However, this is less of an issue with the analyses included in this manuscript, so we are happy to make these changes which we believe strengthens the paper. Of note, a multiracial category was initially included in Table 1, but we did not conduct further analyses with this group due to inability to confidently link this race/ethnicity categorization with census data. For consistency, we now use Black, White, Hispanic, Asian, and Other as our race/ethnicity categorizations throughout the manuscript.***

-       Figures –

o       in the main text, Fig 3., the curves for vaccination by population and for diagnosed cases seem to be pretty equitable – this isn’t commented on. Is this because the analysis is according to the vulnerability index? Would they be more extreme if analysed according proportion in a zip code of Black race/ethnicity?

***Thank you for the opportunity to clarify. For our analyses, we did want to examine different metrics of equity, and didn’t necessarily prioritize one over the other. When interpreting Lorenz curves and Gini coefficients, it is clear that the inequities are most prominent when considering SVI and deaths. However, we would argue that Gini coefficients between 0.1 and 0.2 (which is where they were for population and cases at the end of our observation period) are still quite substantial and do not represent an equitable outcome (although they have improved over time which is also an important finding). This interpretation is further support by the systemic differences evident between zip codes with lower versus higher rates of vaccination relative to population. In response, we provide additional clarification of this interpretation.***

***“When quantifying these disparities using Lorenz curves, we note that disparities in vaccinations were highest relative to population-level social vulnerability and deaths, but still evident—albeit reduced—even when considering vaccinations relative to the overall population and diagnosed COVID-19 cases.”***

o       The curves in the supplementary material change over time – does the same happen in Fig. 3, or is in an average?

***Thank you for the opportunity to clarify. Figure 3 represents the Lorenz curve that includes data on all vaccinations up through February 15, 2022 (previously December 15, 2021). However, it is also an important point of interest to also to see how these have changed over time as equity may have changed over time. Thus, the figures in the supplemental material are data videos that show how the Lorenz curves have changed over time. We have added the following clarification in the Figure legend to indicate that it includes data up through February 15, 2022.***

***“Figure 3: Lorenz Curves of Disparities in COVID-19 Vaccinations. This figure depicts modified Lorenz curve examining disparities in COVID-19 vaccinations as of February 15, 2022…”***

-       Language:

o       Line 70 – ‘recapitulate’ – please reword - not sure what this is supposed to mean

***Thank you for the opportunity to clarify. We have reworded per below.***

***“The initial wave of coronavirus disease 2019 (COVID-19) redemonstrated and highlighted historical inequities in health by race and other social indicators of vulnerability [1-3]…”***

o       Line 76 – ‘minoritized’ – please reword – suggests people are being turned into minorities

***Thank you for the opportunity to clarify. We use the word “minoritized” to draw attention to the fact that these groups have been actively marginalized in society through social processes. This is in accordance with established usage of the term to frame the systemic nature of their experience as minorities (***[***https://www.thelancet.com/pdfs/journals/lanpub/PIIS2468-2667(20)30162-6.pdf***](https://www.thelancet.com/pdfs/journals/lanpub/PIIS2468-2667(20)30162-6.pdf)***). Thus, we would advocate for maintaining “minoritized” given the unique an important framing to provides to the issues we discuss.***

-       ‘Race’ – please consider terminology, using ‘racial and ethnic minority groups’, e.g. as CDC do. (<https://www.cdc.gov/coronavirus/2019-ncov/community/health-equity/race-ethnicity.html>).

***Thank you for the suggestion. We have done so in response to the editorial comments (see above)***

-       Tables – need abbreviations to be explained

***Thank you for identifying this. We have now added abbreviations to the Table footnotes.***


**Reviewer #1**

This is an excellent, well-written article that brings a health equity lens to the rollout of SARS-CoV-2 vaccination in two large cities in Missouri. The data are population-based and the study produced collaboratively by academic researchers and health department staff. Despite COVID-19 vaccines being freely available, how they are made available will affect who gets them quickly enough to derive the most reduced risk in the burden of infection (hospitalizations, long COVID, deaths). The authors contextualize their findings in terms of both historical systemic racism in medicine and public health, as well as how implementation intended to counter inequities did not prevent what ended up being substantial racial/ethnic and social inequity in the uptake of vaccines. I have a few comments and suggestions intended to help improve the quality and impact of their manuscript:

***Thank you very much for the positive feedback.***

1. Is there a way to examine, or at least discuss, vaccine uptake as it relates to timing of major surges. That is, in addition to having lower cumulative vaccination rates, were minoritized and vulnerable communities also less likely to be vaccinated in advance of the delta and omicron surges? This speaks to the timing of vaccination, which is an important dimension. Some of it is alluded to in Figure 1.

***Thank you for the suggestion. In response, we have included supplemental figures (Figure S5) that show the temporal trends in COVID-19 cases and deaths in St. Louis and Kansas City. We considered overlaying this data on Figure 1, but ultimately felt the figure would become too dense to easily interpret. In examining these figures, there does appear to be a small uptick in vaccinations during the Delta wave, but none during Omicron. We also include discussion of temporal trends in relative to the Delta and Omicron waves.***

***“This was followed by rapid decline with smaller upticks at the end of May and then during the Delta wave beginning in July; there was no corresponding uptick in vaccination rates during the Omicron wave beginning in mid-December (Figure 1, Table/Figures S2-S5).***

***And***

***“Booster rates increased starting in October 2021 and peaked in early December at the beginning of the Omicron wave…”***

2. Outcome of vaccine uptake. There has been some concern about the quality of vaccine coverage data as derived from registries (e.g., double counting, denominator issues). For example, vaccine coverage rates well above 100% for some racial/ethnic groups. Related, 2018 census data may not reflect differential in and out migration that occurred in many jurisdictions during the early phase of the pandemic especially. The authors should at least acknowledged this as a potential limitation. But if they did any investigative work around this with their own data, it would be worth highlighting so that other jurisdictions factor these issues in as well.

***Thank you for this comment. We agree that potential issues with the vaccine registry data is one of the major limitations to the study. The dataset we used from Missouri DHSS does undergo significant cleaning and de-duplication and we performed minor additional cleaning of our own. We obtained the data at the individual-level and merged with it with census data to obtain population-level denominators. For this revision, we updated analyses to use age, sex, and race stratified population estimates from the 2020 census data (although SVI and other zip code-level characteristics still use 2018 data). Results did not meaningfully change, but, even with this more up to date data, migration during the COVID-19 pandemic could have influenced the appropriateness of the denominators for several of the analyses.***

***In response, we include this as an additional limitation. This is in addition to the existing discussion of issues with missing and misclassified data.***

***“Third, we used zip code population estimates from the 2020 census data, but true population sizes—and thus the appropriate denominators for some analyses—may have changed since then, particularly due to the well-documented migrations that occurred during the early phases of the pandemic.”***

***Additionally, we do mention the de-duplication and cleaning that was performed by the Missouri Department of Health and Senior Services prior to our obtaining the data.***

3. The authors describe how much of the initial vaccine rollout was through large medical centers and health care providers. They note that pre-existing disparities in health care access barriers would be perpetuated under this model, which is true. But I think it is worth pulling back the lens even more. Getting vaccinated is not health care. It is a preventive action that requires broad reach in communities to achieve the requisite coverage. By definition, focusing on medical centers and the health care system as points of distribution for an intervention that requires broad community reach outside the health care system is in my view one key example of how and why disparities were created early on. The health care system, hospitals, and medical providers have no public health mandate and no management/implementation expertise in this areas. Thus, they are always going to be the wrong choice for the major node of implementation of a large scale public health initiative that by definition must reach those outside the health care system. Suggest a re-framing.

***Thank you for this comment. We agree and have incorporated stronger language into our discussion.***

***“Although large health systems may have been more readily able to overcome logistic issues and provide the robust cold chain needed for mRNA vaccines, they have limited mandates and expertise for implementing large-scale public health initiatives. Even prior to the pandemic, the significant disparities in who accesses care at these health systems and who is outside of them were well-known [30,33]. Physical access, challenges with scheduling (particularly online), disparities in insurance, lack of community partnerships, and mistrust of large institutions that have largely neglected underserved communities often serve as salient barriers to care-seeking in large health systems for individuals from high SVI communities [27,30,34,35]. Vaccination campaigns are a public health strategy that requires broad reach into communities that large health systems did not have and were not designed for; thus, the strategies relying on these systems did not reach the most vulnerable populations essentially by design even though the vaccines themselves were freely available.”***

4. I am having a heard time wrapping my head around how a sample sizes of 32K Hispanic, 38K Asian, and 187K multiracial are too small to produce useful estimates (Discussion, Line 361), especially since they are not samples, but near full enumerations of those populations in each city. Please consider presenting more nuanced groupings than Black-White-Other.

***Thank you raising this important point, please also see our response to the academic editor. In our initial conceptualization of the analysis, we were concerned about small numbers of individual race/ethnicities at the zip code-level. However, this is not an issue for the way the analysis is currently constructed.***

***In response, we now report on Black, White, Hispanic, and Asian race/ethnicities across all analyses. Given heterogeneity in the Multi-racial category (i.e., multiple combinations) in the vaccine data and inability to clearly link this with population census data, we did not extend analyses to this categorization.***

***This is an important change that we think greatly improves the manuscript.***

5. For the SVI variable in Table 1, consider presenting low, medium, and high so readers can appreciate any gradient that may be there.

***Thank you for this suggestion. We now use low SVI (less than 0.333), medium SVI (0.333 to 0.666), and high SVI (greater than or equal to 0.666) categories for zip codes throughout the manuscript. Of note, with this revision, we now include expanded race/ethnicity and SVI categorizations and also examine completion of the primary series and boosters. To accommodate these changes, we now aggregate St. Louis and Kansas City as we felt additional stratification would make the results too dense for interpretation. However, we do still present rates and cumulative incidence of vaccination stratified by region in the supplementary material (Figures S4a and S4b).***

Related, did the authors check that relationships were in fact linear in models where continuous exposures were examined (i.e., in models presented in Table 2)? If so, this should be stated in the methods. If not, the authors should examine these variables with fewer smoothing assumptions (e.g., as 3+ level categorical variables).

***Thank you for the opportunity to clarify. In our initial manuscript, we treated continuous variables as linear for simplicity and interpretability to avoid the results from becoming too dense, but we did not formally assess for linearity. In response for the revision, we did visually confirm that most of the continuous zip code level variables did in fact have linear relationships with vaccination. However, there was some degree nonlinearity for SVI, cases, and deaths. For SVI, we treated it as a 3-level category, which aligns with the rest of our analyses. We opted to maintain cases and deaths as linear since 1) there was only small amounts of nonlinearity to the relationship that we felt were reasonable to average out over variable range, 2) the interpretation did not change, and 3) these variables were not a primary focus of the analysis and presenting them as linear allowed for a more straightforward presentation of the results. We now also mention this check for nonlinearity in the methods.***

**Reviewer 2:**

I mostly confine my remarks to statistical aspects of this paper.  The overall method is sound, but I have a few issues to resolve before I can recommend publication.

***Thank you.***

I will note that it could use some more editing for English usage. There are doubled words (had had), misplaced articles ("endured the disproportionate" should not have a "the"), wrong words ("empiric" should be "empirical") and so on. I don't usually comment on grammar, but here, it affects readability and may affect credibility.

***Thank you for bringing this to our attention. We have performed line by line revisions to address grammatical errors.***

One non-statistical comment: I think the title should reflect that this study was only in two cities in one state in the USA. As is, it sounds universal.

Still on the title, but now statistical: I think it should be "over time" not "and time"   --   disparities across social vulnerability, race, and location, over time.  After all, while it is true that you can be inequitable regarding place, race, etc, it's hard to be inequitable about time -- July isn't going to complain that it got fewer vaccines than April.  Another possibility to end with "controlling for time".

***Thank you for these suggestions. We adjusted the title per editorial comments above to 1) state that the analyses was performed in St. Louis and Kansas City and 2) discuss that it assess disparities “over time”. The current title now reads “Quantifying Inequities in COVID-19 Vaccine Distribution Over Time by social vulnerability, race and ethnicity, and location: A Population-Level Analysis in St. Louis and Kansas City, Missouri”.***

p. 5 line 134  What are "health facilities"?  Are these hospitals?  And you aren't really looking at the size of the facility, but at its vaccination rate. This will surely be related to size, but also to other factors, such as the existence of other facilities nearby. (If a big hospital is near a mass vaccination site, people may go to the latter).

***Thank you for the opportunity to clarify. We refer to health facilities as any location offering routine health car services, which includes hospitals, outpatient clinics, urgent care, emergency room, etc. Although this categorization can be a bit broad, we do attempt to distinguish from other types of sites, such as facilities run by the public health department, pharmacies, employer or school-based vaccination initiatives, and other specialized locations (e.g., dialysis centers, nursing homes, etc.).***

***Although the vaccination volume does likely correlate fairly well with the overall size and patient volume of the facility, the reviewer is indeed correct that the small, medium, and large is specific to vaccination. In response, we have changed the categorization to Small Volume, Medium Volume, and Large Volume Health Facilities. We clarified the Methods as per below.***

***“We categorized vaccine administration sites into health facilities (e.g., clinics, hospitals, health system-affiliated sites) that administered either a small, medium, or large volume of vaccinations (i.e., less than 1000, 1000 to 10,000, or greater than 10,000 individuals vaccinated, respectively)…”***

p. 6 line 154-156  I wouldn't do this, but, since you also treated this as a continuous variable, it's not so bad.

***Thank you for the opportunity to clarify. We were unclear exactly to which analysis the reviewer was referring, but we believe they were commenting on our categorizing zip codes into quartiles based on their position on the Lorenz curves. As standard Lorenz curves are formulated in a way that are agnostic to any zip code-level characteristics, but we performed this analysis to help identify some of the patterns and characteristics that were associated with being on either the left or right side of the curve (i.e., lower or higher rankings for vaccination rates) beyond the color-coded SVI. We do recognize, though, that these categorizations do not necessarily have intrinsic meaning and simply serve as a tool to gain some additional insights, which is why we included those results only in the supplemental material.***

***We do apologize if the reviewer was referring to something else, and we would also be happy to clarify additional issues if we misinterpreted the comment.***

line 160  Rather than % of the population, this should be based on a number of people. ZIP codes are very disparate in population. I know that, in New York City, some ZIP codes have no residents at all (these are large office buildings that have their own ZIP).  In Missouri, ZIP codes can have as few as 6 people (63464) and as many as 75,000 (63376) (See <https://www.missouri-demographics.com/zip_codes_by_population)>. The latter one is actually in your data. Just checking St Louis (because I got interested) 63140 has only 347 people).

***Thank you for raising this point. We agree that the denominator is what is important. In response, we now restrict to zip codes with at least 25 individuals in each race/ethnicity group. Based on responses to other reviewers, we have altered this plot to include primary series and booster completion only relative to population, but compare vaccination rates in Black, Hispanic, and Asian communities relative White communities in the same zip code. Of note, we did assess other thresholds (>50, >100, >200, etc.), but did not find that these higher thresholds was substantially better at removing outliers.***

Despite all the above, I think this is a good paper that uses somewhat unusual statistical methods in a good way.

***Thank you again for the positive feedback.***

**Reviewer 3:**

Summary

Differences in vaccine coverage between populations may result in large disparities in COVID-19 morbidity and mortality. In this paper, the authors assess inequities in COVID-19 vaccine coverage in 7 counties in St. Louis and 4 counties in Kansas City throughout the first year of vaccine availability. The authors specifically look at Black and White populations in these counties and compare vaccination rates relative to the following measures: time, race, zip-code level social vulnerability index (SVI), vaccine location type, and COVID-19 disease burden. The authors summarize inequality using Lorenz curves and Gini coefficients, tools/methods from economics. The key findings from this study are that large inequities in vaccination between Black and White communities were seen early on during vaccine distribution and that inequities only decreased once methods of distribution broadened, and that inequities persist. This is significant because it highlights the importance of public health strategies taking into account structural drivers of inequities.

Strengths

The paper is very well written. The authors do a great job outlining why research on inequities in vaccination rates should look at multiple structural and social determinants to gain a better understanding of how such inequities may be addressed. The methods are well justified and the use of traditional economics methods to describe health inequities is creative. The methods are described clearly in an accessible way that made it so even readers who are not well versed in statistics could understand at a basic level what was done. The graphs and figures are good, but could be made even more clear (details below).

***Thank you for the positive feedback!***

Critiques

1. The paper is an excellent retrospective look at vaccine rollout. However, I expected a little bit more about what cities or counties can do moving forward in the Omicron era. E.g., Figure 1 looks like cumulative proportion of 1 vaccine dose, would it be fairly easy for authors to look at 2 doses and booster? Given where discussion is now with fully vaccinated meaning at least 2 doses and booster availability, do these results hold up? A full look at booster coverage may be out of scope of the paper. However, there is concern about people not getting boosters and people (disproportionately Black, high SVI) who got their vaccine later were not eligible for boosters during the early Omicron wave. Some additional analysis - or discussion - of this point would be helpful to guide policy makers in applying the findings to the current moment.

***Thank you for raising this important comment. We agree with the reviewer. When this analysis was initially conceptualized, we focused on receipt of the initial vaccine dose as a metric of reach of the vaccination strategies (in part because a single dose was still considered to provide substantial protection at the time). Additionally, boosters had just recently been rolled out and we also did not have data during the Omicron period at that time. However, at the time of this revision, this data up through February 15, 2022 is now available to us. We agree with the reviewer also that at this stage of the pandemic, completion of the primary series (and also receiving a booster) is certainly the most meaningful metric for protection against COVID-19 disease given increasing levels of immune escape with newer variants.***

***Thus, in response, we have made significant changes to the manuscript so as to present the most relevant depiction of vaccine equity at this stage in the pandemic. First, we shift the primary focus from receipt of one vaccine dose to completion of the original series and also conduct parallel analyses to examine equity in booster uptake. Second, given the density of information presented, we now aggregate results for St. Louis and Kansas City for the main tables and figures (stratified results are still presented as supplementary information). We believe this is a reasonable choice as differences between these two regions were minimal and stratification yielded limited additional insights (particularly when compared the significant insights gained by examining completion of the original series and boosters).***

***Lastly, we include additional discussion of these results throughout the manuscript, specifically highlighting that patterns of disparities were similar with primary vaccine series rollout and boosters, but the magnitude was often worse with the booster rollout.***

***We believe these changes substantially strengthen the paper and make the findings more relevant to a wider audience. Importantly, these modifications do not qualitatively change results (i.e., receipt of a single vaccine dose was similar to rates of completion of vaccine series). Disparities do worsen a bit, driven by differential completion rates at each stage in the vaccination cascade, and we do still present this data in supplemental Table S2.***

2. I have several suggestions to make the tables and figures even clearer:

-       Across all tables and figures, there should be more clarity around what receipt of the COVID-19 vaccination is. Is it 1 dose or 2? Does it include boosters? Does it mean something different at different age groups? This should be clear in the title or note to each table/figure.

***Thank you for the opportunity to clarify. Per the discussion above, we have now shifted the main outcomes for the paper to be completion of the original series. We clarify this throughout the paper and the tables and figures.***

-       Table 2. The regression coefficients for zip-code characteristics are difficult to compare with each other. I'd suggest standardizing the regression coefficients (dividing by the standard deviation of the predictor) to enable comparisons.

***Thank you for the suggestion. We sought to scale regression coefficients to increments that were contextually meaningful and relevant. We do agree with the reviewer that scaling the coefficients in a similar manner is also important for comparability. In response, we thus changed the scaling so coefficients represent approximately 50% (though not exactly) of the interquantile range for each variable (and mention this in the figure legend). Although there is still some variability in scaling between variables, we believe this helps to strike the right tradeoff between scaling that is contextually meaningful and also comparable.***

-       Fig 1C and 1D yscale should go 0 to 100% if everyone in the denominator is eligible.

***Thank you for this suggestion. We initially truncated the y-axis so more granularity was visible in the figures, though with our inclusion of additional time and race/ethnicity categories in our analysis, this concern is no longer relevant. We agree with the reviewer and have now changed the y-axis to go from 0 to 100%.***

-       Figure 1, the lines all have colors in a similar tone (making it difficult for the colorblind) and making high/low SVI and White/Black comparisons difficult. I recommend changing the colors/patterns to make the graph easier to read. E.g., change lines to two colors, one color for low SVI and another for high SVI, and then solid lines for Black and dashed lines for White. (or something similar) That change would make it much easier to read this graph.

***Thank you for this suggestion; we agree. For Figure 1, we have now use color-coding for race/ethnicity categories and solid vs. dashed lines for SVI. Per comments from reviewer 1, we included additional race/ethnicity categories as well as SVI categories; thus, we have changed the Figure so each panel only includes data on one race. We believe these changes make the figure substantially easier to read.***

-       Figure 2. Font is too small. Consider month abbreviations, consider removing the decimals on the percentages… should be easier to read

***Thank you again for these suggestions. We have included month abbreviations, increased the font size, and removed the decimal points.***

-       Figure 3 - Color coding is interesting and could help people understand intuitively what the Lorenz curves are doing. Where blues and reds are more mixed, the line is closer to diagonal (less inequality) where blues are together and reds are together is where curves are far away from diagonal (more inequality). However, it is rather hard to see colors. Is there a way to make the colors (dots) bigger?

***Thank you for the suggestion. We formulated the Lorenz curve with color-coded line segments as there were too many zip codes to view using individual points. To make it easier to view, we have increased the thickness of the line segments so they are easier to view (although some are still quite small). If necessary, we can increase the thickness even further, though this also obscures some of the “precision” of the line of the Lorenz curve.***

-       Both of the last two graphs are titled "Figure 4".

***We apologize for the oversight. We have corrected this error.***

-       The first "Figure 4". Gini coef is a measure of inequality (not disparity), hence the title should refer to trends in inequality, as measured by Gini coef.

***Thank you for raising this clarification. It is an important distinction and we adjusted it on the figure title and legend.***

-       The first "Figure 4". I find the 4 lines to be distracting. My eye wanted to compare the lines with each other and understand the difference. But the MAIN point of the figure is that the lines fall over time (as indicated in the title of the figure) and that inequity remains high at the end. If the focus is on temporal trends, I think the figure could be improved by showing just the SVI line - and perhaps showing STL and KC on the same plot.

***Thank you for this comment. We agree that one of the main conclusions from this figure is that Gini coefficients end up falling initially, but then either stagnate or fall very slowly during later periods and still remain high across metrics. However, we also did generate this figure to compare how Ginis based on different metrics changed relative to each other. For example, inequities relative to population fell faster compared to cases for the primary vaccine series. For boosters, inequities relative to SVI actually worsened, while the Ginis for other metrics were improving. Based on initial ideas of targeting vaccination efforts based on SVI, it is imaginable that inequities relative to SVI could have improved faster relative to population or cases. The fact that this did not happen and Ginis changed mostly in parallel though is quite telling that targeted efforts to proactively promote equity may not have been very successful in these regions.***

***Thus, we do think it is useful to include all metrics and not just select one to highlight. We do appreciate that the figures may be quite dense so, in response, we have provided additional explanation in the results section to bring out these nuances.***

-       The second "Figure 4" (i.e. Figure 5 - with 8 panels). I think there's too much going on here. I'd encourage the authors to simplify the figure so the main point is clearer. E.g., panels A-D are enough. Panel A/B show that SVI was correlated with vaccine uptake in blacks, but not in whites (HUGELY IMPORTANT FINDING); panel C/D show that blacks are substantially undervaccinated relative to whites when accounting for case rates. Panels E/F - the data are a bit all over the place, maybe a small numbrs issue with zip-level death rates. Panels G/H - I don't understand what "total SVI in black/white population" means. If the point is that vaccine coverage in inversely associated with social vulnerability, then that point has already been made earlier in the paper.

***Thank you for these suggestions. We do agree that the bubble plot figure could be difficult to interpret (and some panels also suffered from small denominators). In response and based on other changes to the manuscript, we have now focused only on the vaccination rate by population version of the figure, but compare vaccination rates in Black, Hispanic, and Asian communities relative to the White communities in the same zip code. We also do this for the primary vaccine series and boosters.***

***Of note, we have also made the x-axis and y-axis for the figures equivalent. The reviewer notes that the SVI seemed to correlate highly with vaccine uptake in black communities, but less so in White communities. After interrogating this further, we realized this was more artifact due to the 16:9 scaling for the figure which made the x-axis longer than the y-axis. We do include***

3. STL vs. KC. There were some notable differences between St Louis and Kansas City counties, but little discussion of these differences. Did the cities take any different approaches that affected vaccine coverage?

***Thank you for this comment. This is an important point. Unfortunately, we are not aware of any systematic differences in the approaches that St. Louis or Kansas City regions took to vaccination, and do not have the local contextual insight to understand whether any particular strategies were more or less successful in a particular area. Any interpretation of the small differences between the regions would be speculation based on regional known differences such as demographics, but far from definitive.***

***Additionally, in response to other comments by reviewers, we have now made changes to the manuscript to 1) include other racial/ethnic groups and 2) examine completion of the primary series and boosters. Given the density of the data, we felt additional stratification by region would make results extremely difficult to interpret. Thus, in this revision, we now aggregate results from St. Louis and Kansas City. Although we lose the extra nuance by region, we think the more complete examination of racial/ethnic groups and primary series/boosters strengthens the paper significantly and will be of more interest to readers (particularly since stratification by region did not yield significant additional insights). Of note, we do still present rates and cumulative incidence of vaccination stratified by region in the supplementary material (Figures S4a and S4b). We hope the reviewers also agree these changes overall strengthen the paper.***

In all, the authors have conducted a timely and important analysis of small area vaccination inequities. Their emphasis on the role of concrete interventions (decentralized vaccine delivery) to mitigate vaccine inequities is a major strength of the paper. The findings will be valuable for policy makers, in addition to pushing the literature forward.

Jacob Bor
