# Supplementary material for: Quantifying inequities in COVID-19 vaccine distribution over time by social vulnerability, race and ethnicity, and location: A population-level analysis in St. Louis and Kansas City, Missouri
Source: PLoS Med. 2022 Aug 26;19(8):e1004048. doi: 10.1371/journal.pmed.1004048 (PMC9417193; doi:10.1371/journal.pmed.1004048)
Supplement: Attachment — Submitted filename: PLOS Med Response to Reviewers2.docx [file pmed.1004048.s017.docx]

***RESPONSE TO REVIEWERS***

**Reviewers #1 and #2**

The authors have been very responsive to reviewer and editorial comments.

The authors have addressed my concerns and I now recommend publication.

***Thank you for very much***


**Reviewer #3:**

The authors have responded to all of my prior concerns in this revision. The manuscript has been strengthened in particular by the full data update (no small amount of work) to include analyses of the full vaccination series and booster coverage. The expanded focus to look at other racial/ethnic groups is welcome, and the decision to de-emphasize the KC vs. STL distinction is reasonable based on the large amount of content presented. The exposition and exhibits are clearer as a result of the suggestions of other reviewers and Editors. The definitions of equity, race/ethnicity, and minoritized are careful and accurate to the U.S. context.

***Thank you for very much.***

The authors should mention explicitly that there were not enough American Indian / Alaska Native persons in the population to be included as a separate category with statistically stable estimates. (There is some understandable consternation in the AIAN research community about being lumped into "Other" without justification.) I have no further critiques of this fine paper.

***Thank you for this comment. This is an important point. We made changes to the limitation section to be more specific as to why our racial/ethnic minority categories were limited.***

***“Fourth, in this analysis we were unable to provide more granular details and include separate categories for other racial/ethnic minorities such as indigenous or multi-racial individuals, either due to small populations in the regions that would lead to unstable statistical estimates or the inability to link these population across data sources.”***
